# Steering interfacial molecular structures by substrate–molecule charge transfer

**DOI:** 10.1039/d6cp03153f

**Published:** 2026-11-03

**Authors:** Benjamin Achatz, Matthias Zeilerbauer, Marco Thaler, Milan Ončák, Shadi Fatayer, Laerte L. Patera

**Affiliations:** a Department of Physical Chemistry, University of Innsbruck 6020 Innsbruck Austria benjamin.achatz@uibk.ac.at laerte.patera@uibk.ac.at; b Department of Ion Physics and Applied Physics, University of Innsbruck 6020 Innsbruck Austria; c Physical Science and Engineering Division (PSE), King Abdullah University of Science and Technology (KAUST) Thuwal 23955-6900 Saudi Arabia

## Abstract

Intermixed self-assembled monolayers (SAMs) provide a powerful approach for tuning inorganic–organic interfaces, but their local structure and energetics often remain elusive. Here, an intermixed stripe phase of fullerene C_60_ and Zn(ii)–5,10,15,20-tetrakis(4-aminophenyl)porphyrin (Zn–TAPP) on a Ag(111) surface is identified, establishing a direct link between molecular assembly and charge transfer. Scanning tunnelling spectroscopy (STS) and Kelvin probe force spectroscopy (KPFS) have been used to map electronic states and local contact potential differences (LCPD). Compared to close-packed C_60_ islands, the stripe phase exhibits a larger interfacial dipole and increased occupation of previously unoccupied C_60_ states, consistent with enhanced charge transfer from Ag(111) to C_60_. Analysis indicates that larger C_60_ spacing in the stripe geometry reduces Coulomb repulsion, stabilizing the intermixed phase and enhancing charge transfer with respect to close-packed domains. These results provide direct evidence of charged C_60_ molecules in stripe structures and guidelines for engineering intermixed SAMs with tailored interfacial electronic properties.

## Abstract 2

Porphyrin-templated C_60_ stripes on Ag(111) enhance substrate-to-molecule charge transfer by reducing lateral Coulomb repulsion, demonstrating that nanoscale molecular packing can be used to engineer interfacial electronic properties.

## Graphical abstract

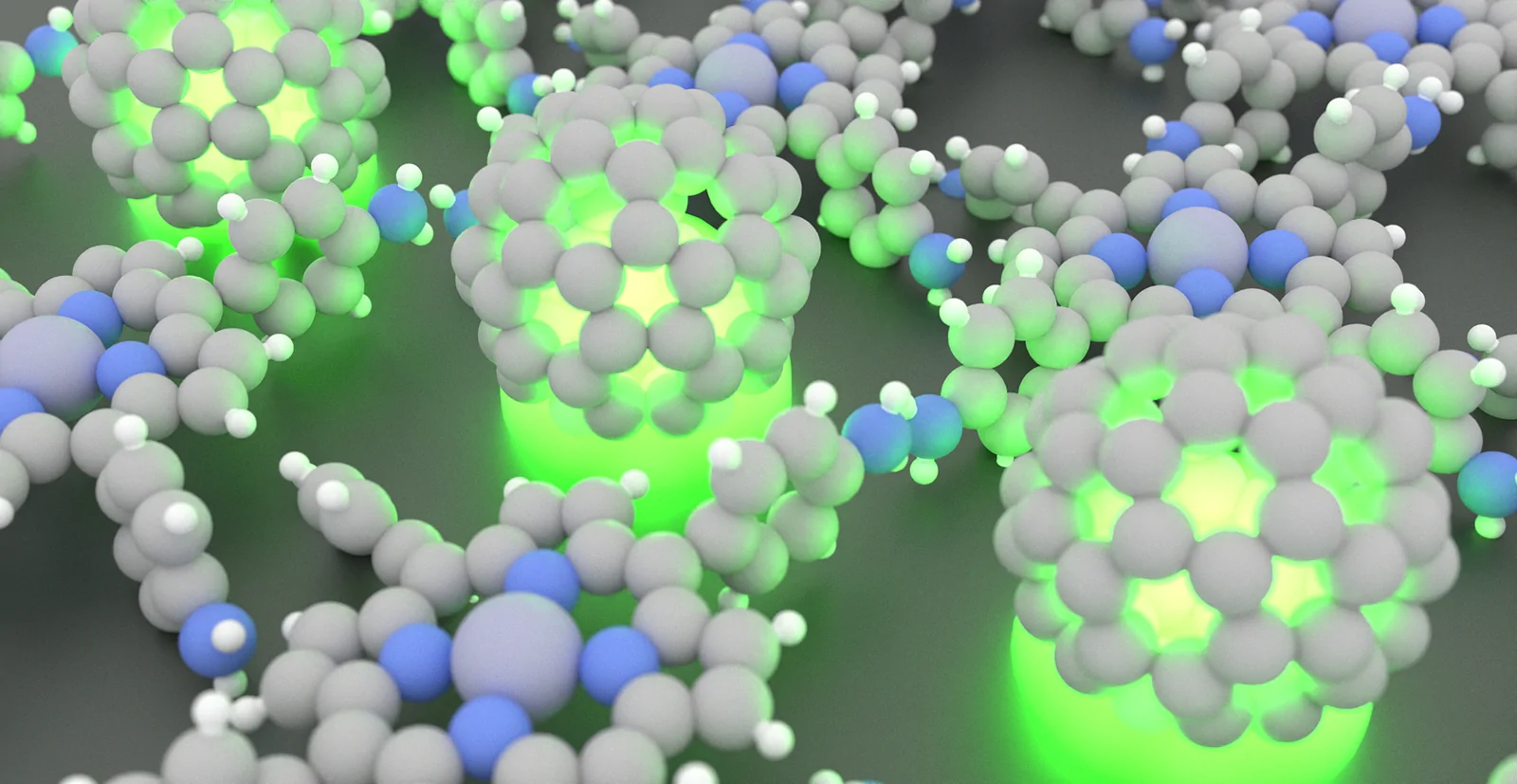

## Introduction

Organic electronic devices have emerged as a promising alternative to conventional inorganic semiconductor technologies. Their appeal lies in the potential for cost and energy efficient fabrication, including solution processing and low temperature deposition.^1,2^ The chemical tunability of organic molecules enables devices with tailored electronic and optical properties across a wide range of applications.^1,3^ Device performances have been improved by tuning both the molecular components^4^ and the metal electrodes,^5^ with particular attention on interfacial energetics and charge injection barriers at the metal–organic interface.^6^ Among various strategies to control this interface, the usage of self-assembled monolayers (SAMs) has proven especially powerful.^7–9^ Intermixed SAMs allow for precise modulation of work function, local dipoles, and contact resistance, thereby improving device efficiency^10,11^ and transport properties.^12^ Realizing this potential requires a molecular level understanding of the intermolecular interactions and packing rules that govern the formation of such intermixed SAMs.

Investigations of SAMs and metal–organic interfaces have relied extensively on surface-sensitive spectroscopic techniques such as X-ray photoelectron spectroscopy (XPS) and ultraviolet photoelectron spectroscopy (UPS).^11,13,14^ These methods enable the determination of composition, bonding, level alignment, and average molecular orientation. However, these techniques inherently perform spatial averaging over the illuminated area, thereby masking the molecule-scale heterogeneity and local physicochemical properties that are intrinsic to intermixed SAMs.^15^ Conversely, scanning probe microscopy (SPM) techniques enable direct, local interrogation of structure and energetics at the nanometre scale.^16,17^ Scanning tunnelling microscopy and spectroscopy (STM/STS) provide molecular scale information on packing, defects, and electronic states of SAMs.^18–20^ Non-contact atomic force microscopy (nc-AFM) can be exploited to precisely investigate the chemical structure,^21^ adsorption configurations^21,22^ and adsorption height with sub-angstrom resolution.^23,24^ Additionally, Kelvin probe force spectroscopy (KPFS) probes local contact potential differences (LCPD), enabling the mapping of local surface dipoles with sub-nanometre scale resolution.^25,26^

The realization of intermixed phases is of special interest for optoelectronic devices based on electron donor–acceptor (D–A) pairs,^27^ where, upon photoexcitation, charge separation occurs at the heterojunctions.^28^ Device efficiency can be enhanced by increasing the interfacial area between donor and acceptor domains^29^ and controlling the local structure of the interfaces.^28^ Metalloporphyrins and C_60_ are commonly used as an electron D–A pair due to the formation of stabilized charge-separated states.^30,31^ Intermolecular interaction in bulk heterostructures of porphyrins and C_60_ are dominated by strong van der Waals interactions.^31,32^

Intermixed SAMs of prototypical D–A pairs such as Zn–porphyrins and C_60_ have been reported on a Ag(110) surface, where coverage dependent pore and stripe phases emerge.^33,34^ Stripe formation of C_60_ on a graphite surface has previously been attributed to the interplay between attractive short-range van der Waals interactions and long-range dipole–dipole repulsion between charged C_60_ molecules,^35^ similar to the behaviour reported for rubrene chains on Au(111).^36^ However, direct experimental evidence for molecular charging within these C_60_ stripe phases has so far been lacking.

Here, we report the on-surface synthesis of an intermixed stripe phase of C_60_ and Zn(ii)–5,10,15,20-tetrakis(4-aminophenyl)porphyrin (Zn–TAPP) on the close packed Ag(111) surface. We show that the combination of KPFS and STS enables the experimental investigation and comparison of the degree of substrate–molecule charge transfer for C_60_ in close-packed islands and in the intermixed stripe phase. KPFS provides insights into variations of the local interfacial dipoles, resulting from substrate–molecule charge transfer.^37,38^ STS can be used to elucidate relative shifts in unoccupied-states near the Fermi level (*E*_F_), consistent with partial charge transfer to C_60_.^39,40^ Linking the experimentally determined degree of charge transfer to the structure of the distinct phases provides evidence for the crucial role of electrostatics in the formation of these intermixed SAMs on metal surfaces.

## Results

### Assembly transformation upon annealing

A sub-monolayer of C_60_ and Zn–TAPP molecules has been obtained by deposition on a clean Ag(111) substrate held at 300 K. Post-annealing up to 415 K for 2 h results in the phase separation into large domains of C_60_ and Zn–TAPP (see Fig. 1a). The observed phase separation is consistent with the strong van der Waals interactions reported for C_60_ molecular crystals, making segregation favourable.^41^ On closed-packed transition metal surfaces C_60_ forms hexagonal islands,^42–45^ which nucleate primarily at step edges.^46^Fig. 1b displays the characteristic bright and dim contrasts of C_60_ molecules adsorbed on Ag(111).^42–45^ The dim contrast has been attributed to the formation of single-atom vacancies underneath the respective C_60_ molecules, steered by annealing to 415 K.^42–46^ STM images indicate that approximately 33% of C_60_ molecules adsorb on reconstructed sites (*i.e.* dim configuration). On Ag(111), C_60_ form a (2√3 × 2√3)R30° superstructure (Fig. S1) with centre-to-centre distances of 1.00 nm.^44^ The STM appearance of individual C_60_ molecules reflects their specific adsorption geometry:^43–45,47,48^ adsorption on a 6-membered ring results in 3-fold symmetry and adsorption on a bond between two 6 membered rings leads to 2-fold symmetry.^47^ Ordered Zn–TAPP domains are observed to form preferentially at the centre of Ag(111) terraces. Fig. 1c presents a two-dimensional rectangular lattice of Zn–TAPP (lattice parameters: *a* = 1.68 nm, *b* = 1.61 nm, *α* = 87°).

**Fig. 1:**
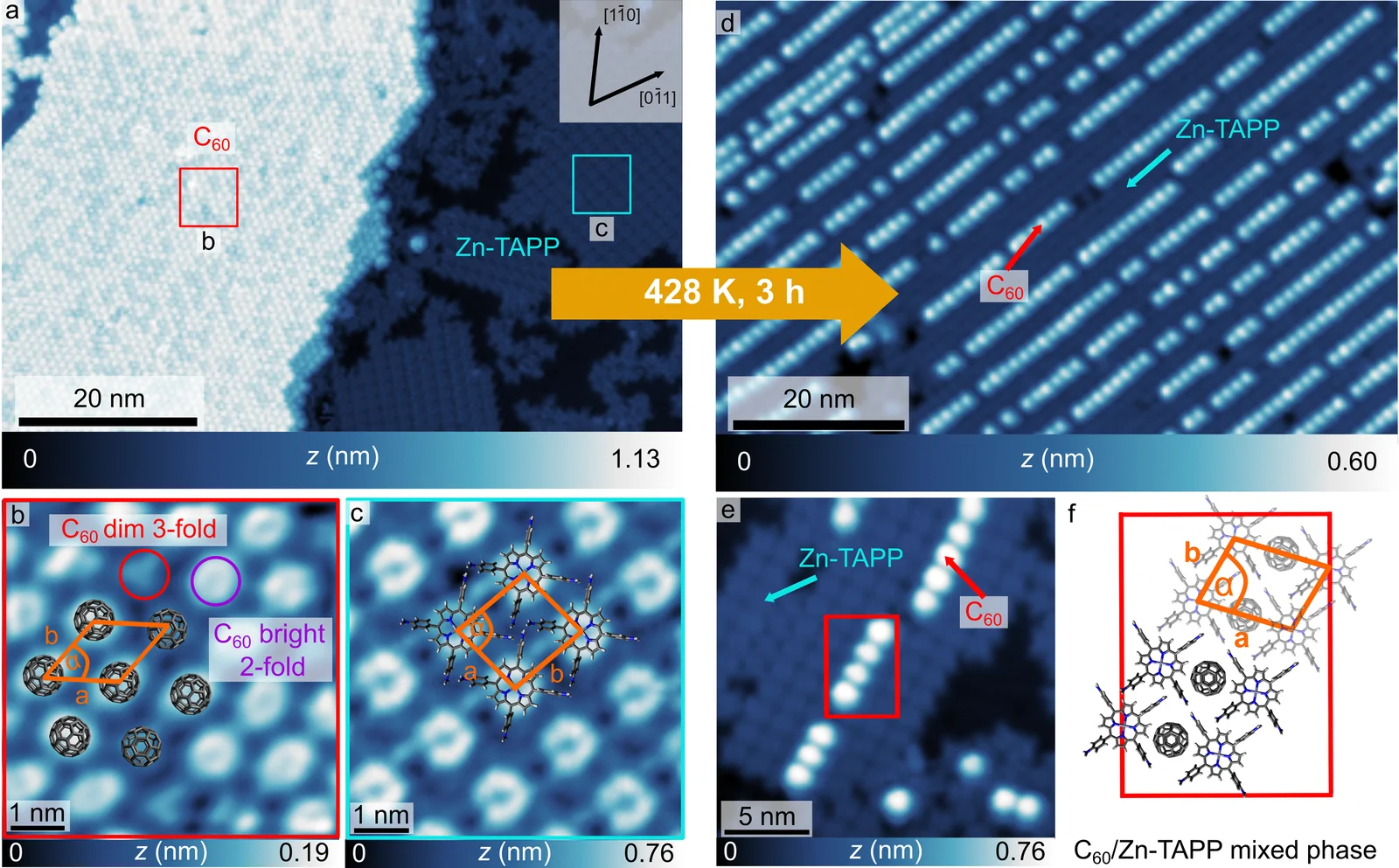
Formation of an intermixed self-assembled monolayer on Ag(111). (a) Constant-current STM image acquired upon deposition of C_60_ and Zn–TAPP on Ag(111) and subsequent annealing to 415 K for 2 h. (b) and (c) High-resolution STM images of ordered Zn–TAPP and C_60_ phases, respectively, overlayed with structural models. (d) and (e) Constant-current STM images acquired after annealing to 428 K for 3 h. (f) Model of the intermixed phase of C_60_ and Zn–TAPP. Unit cells are indicated in orange. Measurement parameters: (a) tunneling current *I* = 20 pA, sample bias voltage *V* = 0.5 V. (b) and (c) *I* = 10 pA, *V* = 0.5 V. (d) *I* = 5 pA, *V* = 1.0 V. (e) *I* = 10 pA, *V* = 0.6 V.

Annealing to 428 K for 3 h results in the formation of an intermixed phase (Fig. 1d and e), where C_60_ molecules arrange in one-dimensional stripes constrained by a surrounding ordered Zn–TAPP array. The structural model of the intermixed phase shown in Fig. 1f reveals pores formed between four Zn–TAPP molecules, with each pore accommodating a single C_60_ (unit cell parameters: *a* = 2.04 nm, *b* = 1.47 nm, *α* = 77°), similar to the structure reported on Ag(110).^46^ The extracted intermolecular distances of C_60_ for the intermixed phase (1.47 ± 0.02 nm) are considerably larger than those in the close-packed phases (1.00 ± 0.03 nm). Several stripe orientations are observed (see Fig. S2). These orientations arise from the presence of differently oriented Zn–TAPP domains on the Ag(111) substrate. The orientational distribution of the intermixed phase is therefore mainly associated with the domain structure and molecular packing of the Zn–TAPP template.

### Kelvin probe force spectroscopy

For isolated C_60_ molecules adsorbed on an unreconstructed Ag(111) surface, density functional theory (DFT) calculations predict charge transfer of up to 0.5 electrons per molecule from Ag to C_60_.^49^ On reconstructed sites, due to the larger hybridization with the substrate, an even larger degree of charge transfer can be expected.^39,44^ The resulting negative charging of the C_60_ molecules introduces significant intermolecular repulsion, potentially disfavouring dense packing and instead promoting the formation of energetically favourable linear arrangements. To elucidate this point, KPFS has been performed to probe the degree of substrate–C_60_ charge transfer. Individual spectra have been acquired on top of C_60_ molecules both in the islands and in the intermixed phase, as well as on Zn–TAPP molecules and on the Ag(111) substrate (see Fig. 2a). A parabolic behaviour of the frequency shift (Δ*f*) upon sweeping the sample bias voltage (*V*) is observed for all spectra in Fig. 2b. The sample bias voltage corresponding to the maximum of the parabola (V*) is defined as the local contact potential difference (LCPD). For *V* = LCPD, the electrostatic contribution to the force gradient is minimized. As a result, the corresponding Δ*f* value (Δf*) mostly reflects van der Waals contributions.^25,26,50^

**Fig. 2:**
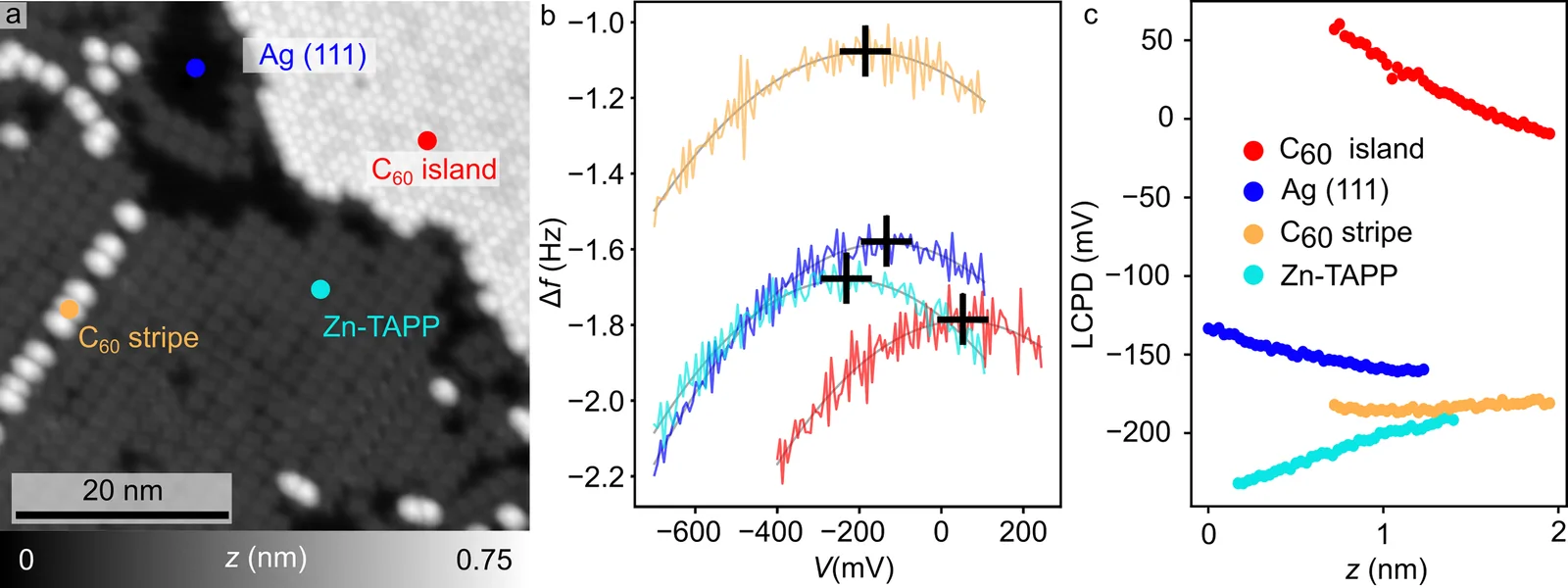
Height-dependent KPFS. (a) Constant-current STM image acquired after annealing to 428 K for 3 h. (b) KPF spectra acquired at the positions marked in (a): on a close packed C_60_ island (red), on a C_60_ in a one-dimensional stripe (orange), on a Zn–TAPP island (cyan) and on Ag(111) (blue). Parabolic fits and corresponding parabola peaks are indicated for each spectrum. (c) Fitted LCPD plotted as a function of the tip height *z*. The LCPD(*z*) curves were offset by the corresponding apparent heights obtained from (a). Measurement parameters: (a) *I* = 20 pA, *V* = 0.5 V. (b) Feedback opened on the marked spots at *I* = 5 pA, *V* = 0.6 V, *z* = 0.3 nm. (c) Feedback opened on the marked positions at *I* = 5 pA, *V* = 0.6 V. Positive *z* values correspond to a tip height increase compared to the STM setpoint. AFM oscillation amplitude *A* = 0.3 nm.

In KPFS, the LCPD value is influenced both by the atomic structure of the tip apex as well as by the tip–sample separation, owing to spatial averaging effects.^25,26^ Consequently, meaningful comparisons of LCPD shifts must be carried out using the same probe and constant tip–sample distances. To disentangle electrostatic effects from height-related artefacts, KPFS measurements should be performed at several tip heights.^25,26^

To examine the LCPD dependence on the tip height (*z*), the tip was stabilized at a defined STM setpoint, the feedback was disabled, and the tip was retracted in discrete height steps. The LCPD(*z*) curve measured above the C_60_ island exhibits a more pronounced increase in LCPD compared to the one acquired on Ag(111). This effect indicates a larger local surface dipole on the C_60_ islands than on the substrate, being induced by an accumulation of negative charge on the C_60_ islands, consistent with previous DFT studies.^49^ In contrast, the lower LCPD(*z*) values observed for the Zn–TAPP domain compared to Ag(111) is attributed to the push-back effect, which suppresses electron spill-out at the interface.^51–53^ Notably, the LCPD(*z*) curve for the linear C_60_ stripe is rather flat, suggesting a smaller surface dipole compared to the C_60_ island. These trends are also observed when plotting the LCPD against Δf* (see Fig. S4). Fig. 3a shows a C_60_ island adjacent to a C_60_ stripe. The corresponding KPFS map (Fig. 3b) indicates that the LCPD of the C_60_ molecules in the stripe phase is lower than the value found for the C_60_ islands.

**Fig. 3:**
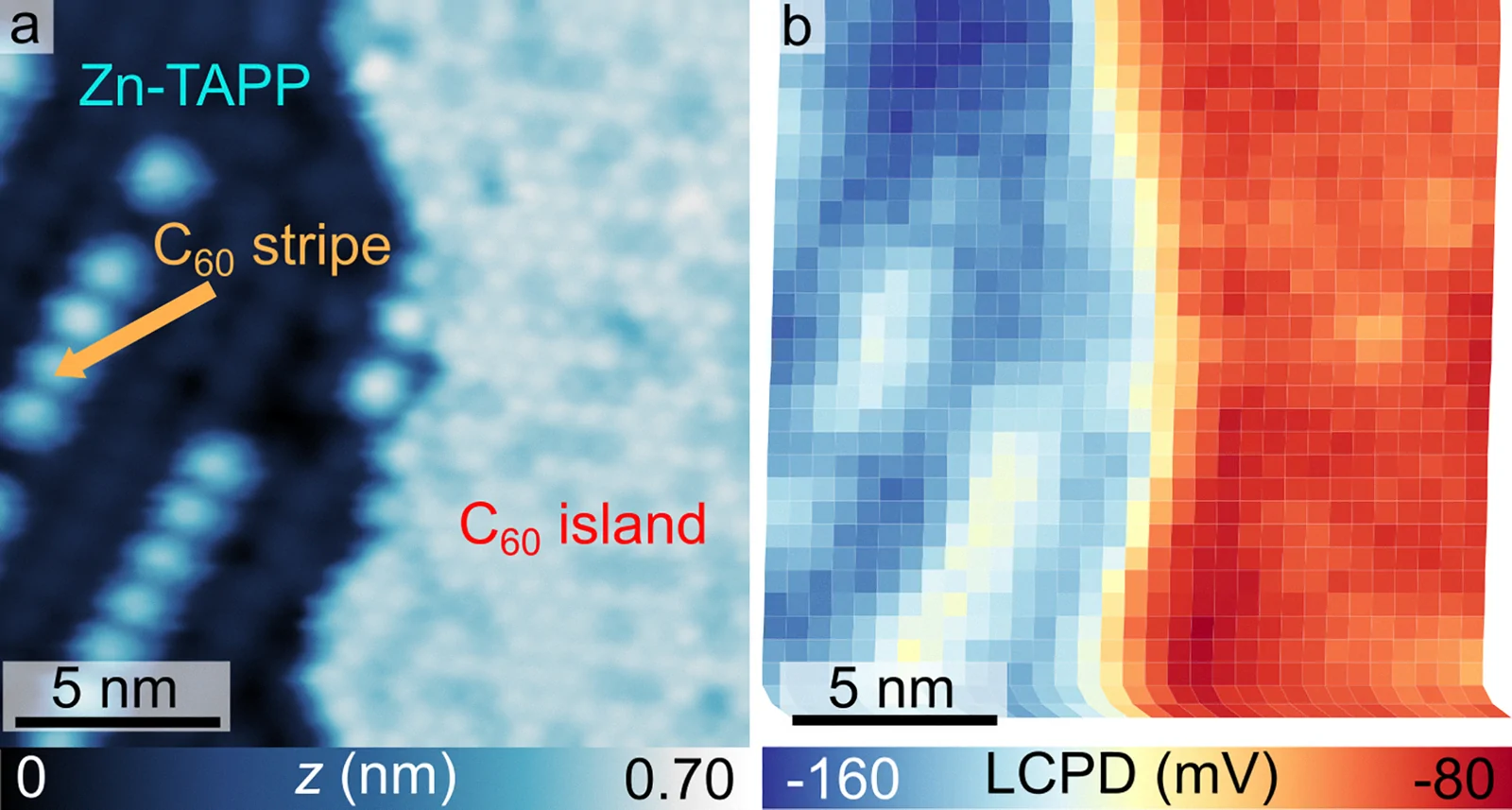
KPFS mapping. (a) Constant-current STM image and (b) corresponding drift-corrected LCPD map of a C_60_ island (right) and the intermixed C_60_/Zn–TAPP phase (left). Measurement parameters: (a) and (b) *I* = 5 pA, *V* = 0.6 V. (b) *z* = 0.3 nm, *A* = 0.3 nm.

It is important to note that an unambiguous assignment of molecular charge states based on LCPD measurements on metal-supported systems is nontrivial. The reduction of the surface dipole due to the push-back effect can partially mask the LCPD increase expected from negative charge accumulation.^54^ Nevertheless, a qualitative comparison remains possible, particularly by considering trends in the LCPD(*z*) curves. In this context, the KPFS data shown in Fig. 2 and 3 suggest a pronounced negative charge accumulation for molecules within the islands, whereas those in the stripe phase seem to exhibit only marginal, if any, charge transfer. However, since the C_60_ islands and stripe phases differ in their lateral dimensions, the lower measured LCPD values for the stripes may also be influenced by the finite lateral resolution of KPFM, which leads to spatial averaging effects.^55^

To better address the effect of dimensionality on the KPFS contrast, we acquired spectra along the Zn–TAPP–C_60_ island interface and across a C_60_ stripe (Fig. 4a), enabling an evaluation of the spatial resolution of the KPFS measurements. As shown in Fig. 4b, the island region exhibits a larger absolute LCPD value. However, the boundary between the C_60_ and Zn–TAPP phases appears significantly smeared out. When considering the LCPD profile across a C_60_ molecule in a stripe, the full width at half maximum (FWHM) is broader (2.1 nm) than the actual size of a C_60_ molecule (≈0.7 nm). This indicates that the KPFS spatial resolution is insufficient to resolve the intrinsic LCPD of the one-dimensional stripes accurately, as substantial averaging with the surrounding Zn–TAPP layer occurs. The lateral resolution of KPFS also further decreases with increasing tip-height as shown for the edge of a C_60_ island in Fig. S5. Overall, the averaging effects reduce the contrast on the one-dimensional intermixed phase, underrepresenting their actual local charges. To attempt to retrieve the actual LCPD value of C_60_ in the stripe phase, we adopt the concept of a point spread function (PSF).^56^ By attributing a Gaussian PSF to the KPFS measurements shown in Fig. 4b, the measured LCPD contrast can be deconvolved (see Fig. S6 and S7). This analysis suggests that the LCPD contrast of the C_60_ in the stripe phase is underestimated by a factor of 7.8 ± 0.6, suggesting that the actual degree of charge transfer for the C_60_ molecules in the stripe phase is significantly larger than directly inferred from the measured LCPD contrast (see Fig. S9).

**Fig. 4:**
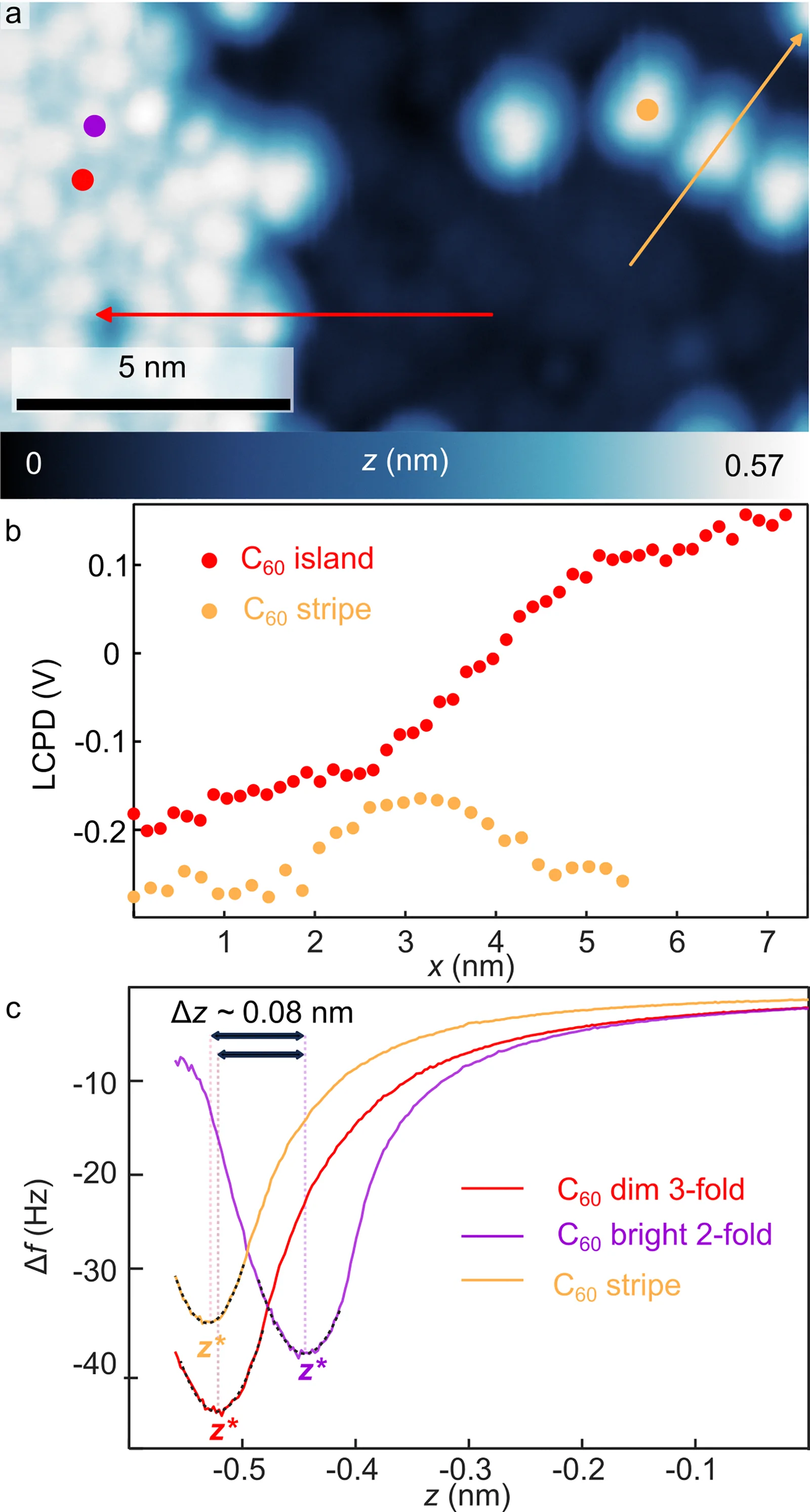
KPFS and Δ*f*(*z*) characterization of the C_60_ species. (a) Constant-current STM image of a C_60_ island and the intermixed C_60_/Zn–TAPP phase. (b) LCPD acquired at constant height along the paths indicated by the arrows in (a): across the edge of a C_60_ island (red) and a C_60_ stripe (orange). (c) Δ*f*(*z*) spectra acquired on different C_60_ species (positions marked in (a)). Measurement parameters: (a) *I* = 10 pA, *V* = 0.25 V. (b) Feedback opened at *I* = 10 pA, *V* = 0.25 V, *z* = 0.0 nm, *A* = 0.1 nm. (c) Feedback opened at *I* = 10 pA, *V* = 0.25 V, *z* = 0.0 nm; Δ*f*(*z*) spectra acquired at *V* = 0 V, *A* = 0.1 nm.

Notably, the LCPD profiles in Fig. 4b show no evidence of positive charge located at the Zn–TAPP molecules at either interface with C_60_, indicating that the negative charge accumulation observed on C_60_ is likely due to charge transfer from the substrate rather than from neighbouring Zn–TAPP molecules.

### Δ*f*(*z*) spectroscopy measurements

Site-specific Δ*f*(*z*) spectroscopy (Fig. 4c) has been performed to determine relative height differences between the C_60_ molecules in the distinct phases on Ag(111). The relative shifts of the tip height *z* corresponding to the turnaround point of the Δ*f*(*z*) signal (z*) provide the height differences. Relative height difference (Δ*z*) of around 0.08 nm between the dim 3-fold and bright 2-fold molecules can be attributed to adsorption of the dim molecules on reconstructed sites.^43,45^ While Δ*z* values were found to be slightly tip dependent, varying between 0.05 and 0.10 nm, the qualitative trends were consistent for different tips. The spectra shown in Fig. 4c were likely obtained with a C_60_ functionalized tip.^53^ Notably, Δ*f*(*z*) curves recorded on C_60_ molecules in the intermixed phase exhibit a z* value close to the one of the dim 3-fold sites, suggesting adsorption on reconstructed sites.

### Characterization of electronic properties with tunnelling spectroscopy

Tunnelling conductance (d*I*/d*V*) spectra acquired for C_60_ molecules in the islands (Fig. 5a) and in the intermixed phase (Fig. 5b) exhibit electronic states close to the Fermi level (*E*_F_), attributed to the lowest unoccupied molecular orbitals (LUMOs) of the C_60_ molecules (Fig. 5c–e).^47^ While gas-phase C_60_ exhibits three-fold degenerate LUMOs,^46,57^ adsorption on metal substrates results in the degeneracy lifting due to hybridization.^44,57^ Distinct peak shapes of the C_60_ LUMO in Fig. 5c–f can be thus attributed to peak splitting and broadening stemming from different degrees of hybridization with the underlying substrate, as well as from π–π orbital hybridization between neighbouring C_60_ molecules,^44^ consistent with observations in thin-film systems.^58^

**Fig. 5:**
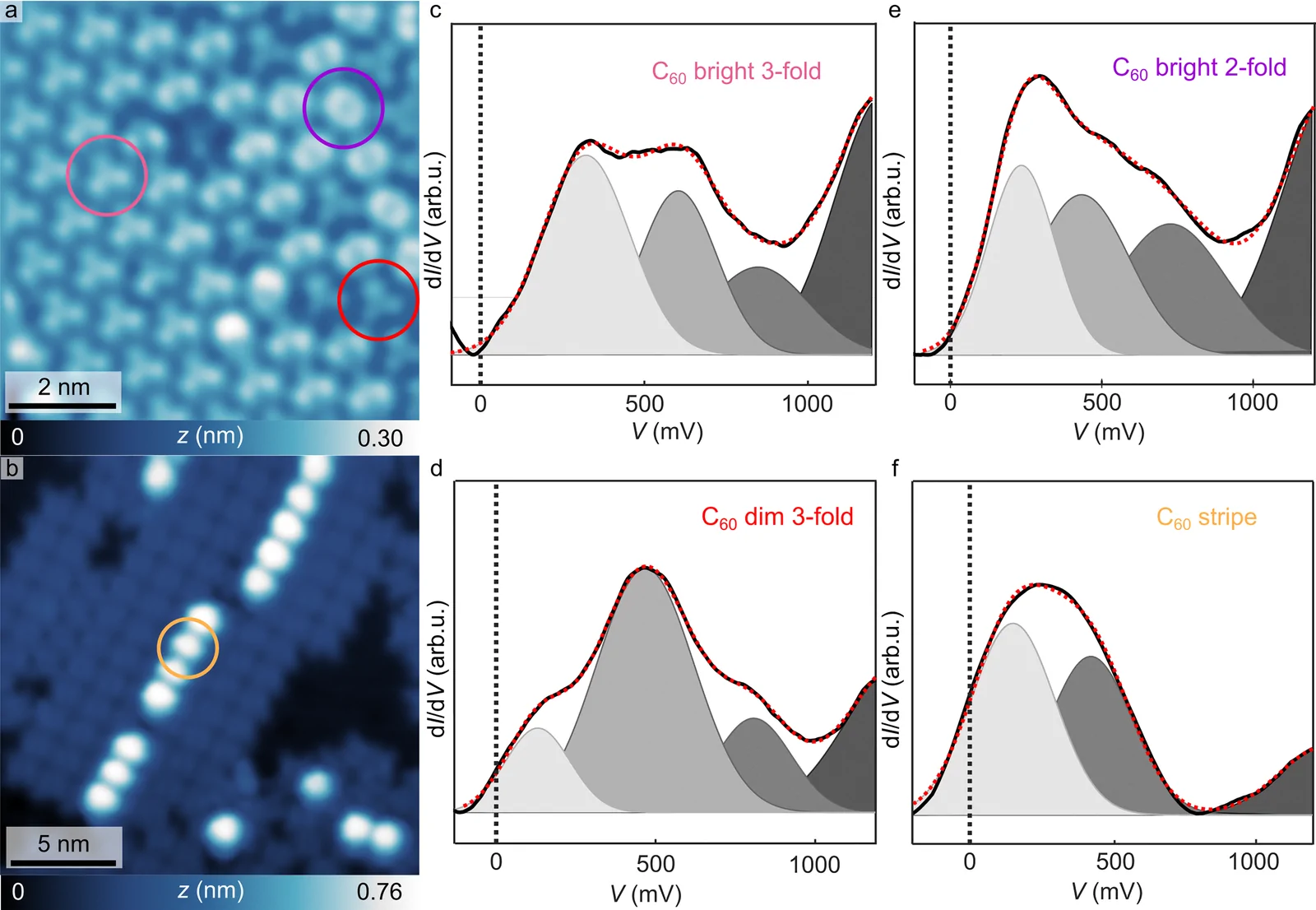
d*I*/d*V* spectroscopy of the C_60_ species. (a) Constant-current STM image of different adsorption geometries within a C_60_ island. C_60_ bright 3-fold, C_60_ dim 3-fold and C_60_ bright 2-fold are highlighted in pink, red and purple, respectively. (b) Constant-current STM image of the intermixed C_60_/Zn–TAPP phase. C_60_ stripe is highlighted in orange. (c)–(e) d*I*/d*V* spectra acquired on C_60_ bright 3-fold (c), C_60_ dim 3-fold (d) and C_60_ bright 2-fold (e). (f) d*I*/d*V* spectrum acquired on C_60_ in the intermixed phase. Measurement parameters: (a) *I* = 10 pA, *V* = 0.2 V. (b) *I* = 10 pA, *V* = 0.6 V. (c)–(e) Feedback opened at *I* = 19 pA, *V* = 1.2 V. (f) Feedback opened at *I* = 10 pA, *V* = 1.2 V. The curves in (c)–(f) are fitted with 3 Gaussian for the C_60_ LUMO and an additional Gaussian for the LUMO+1 (parameters are given in table S1). Areas of the Gaussians are indicated in grey scale and the resulting fits with dotted red lines. (c)–(f) Sample bias voltage modulation *V*_mod_ = 0.04 V_rms_.

The apparent degree of charge transfer has been estimated by determining the area of the LUMOs lying below *E*_F_ (see below). The d*I*/d*V* spectra were fitted with three Gaussian functions for the LUMOs^57^ and an additional Gaussian for the onset of the LUMO+1 (see SI, Table S1). To determine the total areas of the LUMO orbitals the contributions of the three fitting components (between *V*_min_ = −0.2 V and *V*_max_ = 1.2 V) were summed up. The degree of charge transfer (*n*_e^−^_) can be estimated by calculating the apparent LUMO occupation, assuming full occupation of the three LUMOs corresponds to six electrons per molecule (see SI Table S2):1
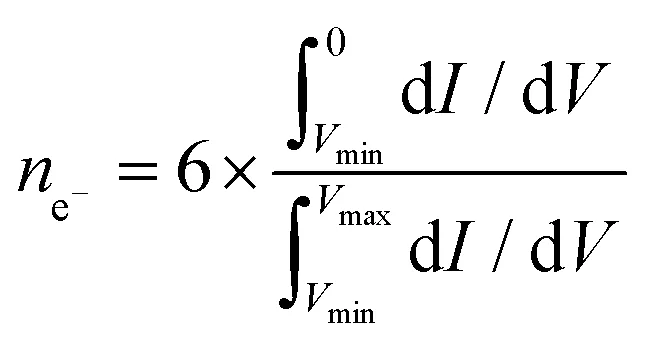
Minimal crossing of *E*_F_ in spectra of bright 3-fold and 2-fold C_60_ indicates negligible charge transfer (0.04 and 0.03 electrons per molecule, respectively). A slightly higher value is calculated for dim 3-fold C_60_ molecules (0.08 electrons per molecule), although this still corresponds to a relatively low level of charge accumulation. In contrast, an increased degree of charge transfer of 0.50 electrons per molecule was determined for C_60_ molecules in the intermixed phase, suggesting that the one-dimensional phase enables a larger degree of charge transfer with respect to the dense phase.

Interpretation of energy shifts in d*I*/d*V* spectra requires a detailed understanding of the energy alignment at the metal–organic interface.^57,59,60^ It is important to note that, for molecules adsorbed on metal surfaces, d*I*/d*V* spectroscopy does not provide the actual energy alignment of frontier orbitals. In electron spectroscopies, charge injection and removal are associated with an additional energy cost, commonly referred to as the Hubbard on-site energy, which leads to an apparent widening of the HOMO–LUMO gap for isolated molecules (see C_60_ (gas) in Fig. 6).^57^ Upon adsorption on a metallic substrate the injected charge is screened by an image charge within the substrate (Δ*E*_pol.(sub.)_ in Fig. 6), reducing the Hubbard on-site energy.^57^ Moreover, a reduction of the surface dipole caused by the push-back effect results in a downwards shift of the vacuum level and of all molecular orbitals (Δ*E*_p.b._ in Fig. 6).^52^ The energy alignment of the frontier orbitals is further governed by hybridization with the substrate, which depends on the substrate reactivity, the adsorbate electronic structure, and its adsorption height.^52^ In d*I*/d*V* measurements, hybridization results in splitting and broadening of the observed peaks.^44,57^ Further broadening and splitting can arise from intermolecular interactions within molecular assemblies.^44,58^ In some cases, this broadening may even lead to *E*_F_ crossing and partial charge transfer between the substrate and the molecular orbitals.^52,61,62^ The additional charge density localized in the molecule arising from the charge transfer from/to the substrate is further stabilized by image charge screening in the metal substrate, leading to an additional downward shift of the molecular levels (Δ*E*_pol.(CT)_ in Fig. 6). The downward shift is counterbalanced by an increase in the local work function induced by the interfacial dipole associated with charge transfer (Δ*E*_μ(CT)_).^52^ This effect shifts the vacuum level upward relative to the molecular states, contributing to orbital pinning near the *E*_F_.^52,62^ In the case of Fermi level pinning, energy shifts caused by screening of the transferred charge and the local work function can therefore not be disentangled. Ultimately, the observed orbital alignment in d*I*/d*V* spectra reflects an equilibrium between image charge screening, hybridization, push-back effects, and dipole-induced vacuum level shifts.^52^ In contrast, eqn (1) is based on the assumption that the d*I*/d*V* curves acquired by tunnelling spectroscopy directly reflect the local density of states (LDOS). The obtained degree of charge transfer (*n*_e^−^_) should therefore not be interpreted quantitatively, but rather serves as an estimate for relative comparison between different C_60_ species.

**Fig. 6:**
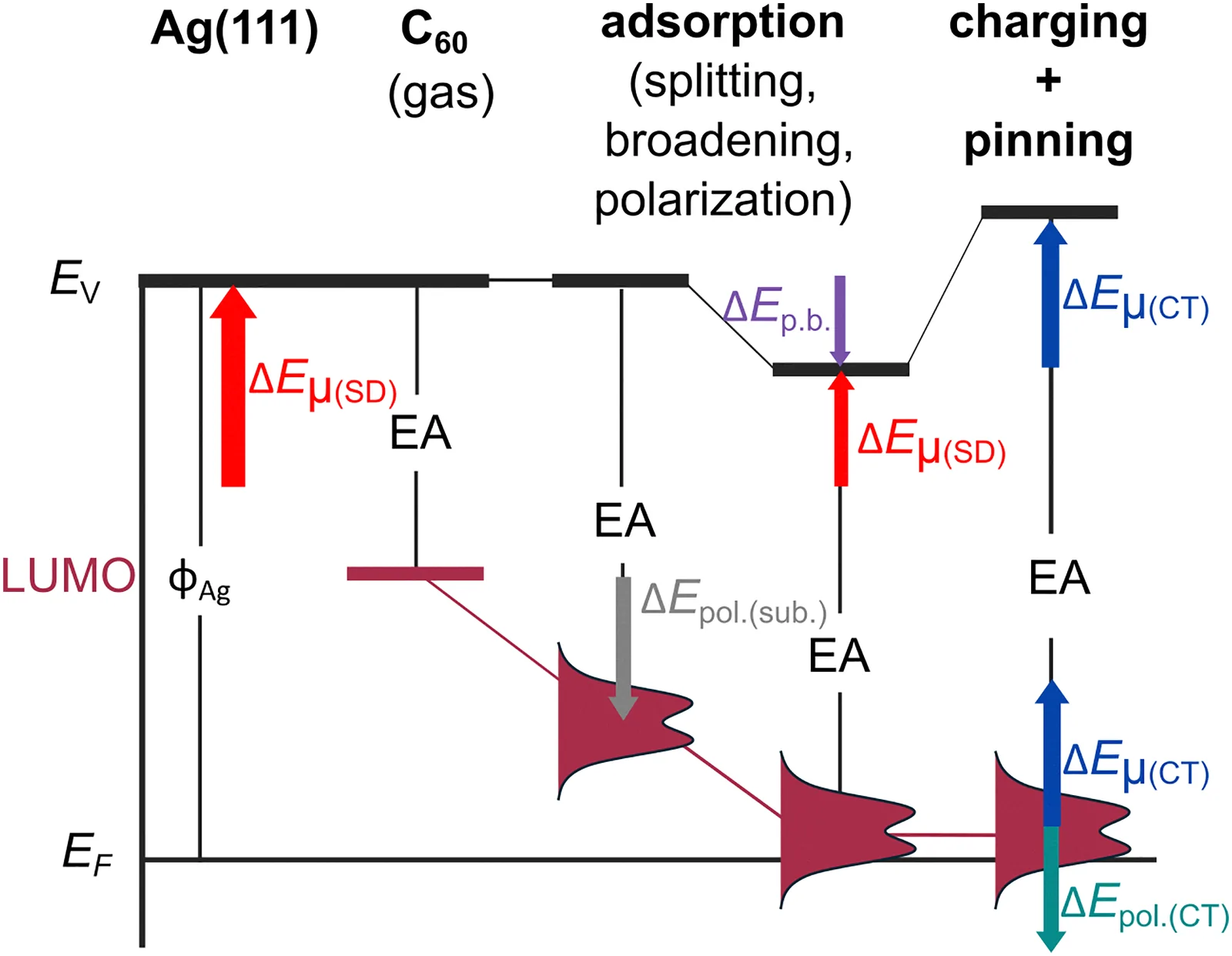
Energy alignment of the C_60_ LUMOs upon adsorption on Ag(111). Bold black lines show the relative position of the vacuum level influenced by changes in the local surface dipole (red and blue arrows). Alignment and peak shape of the C_60_ LUMOs is shown in dark red. The contributions of the push-back effect (Δ*E*_p.b._), the polarization by the substrate (Δ*E*_pol.(sub.)_), the surface dipole (Δ*E*_μ(SD)_), the additional screening of the transferred charge (Δ*E*_pol.(CT)_) and the induced electrical dipole (Δ*E*_μ(CT)_) are indicated with arrows.

## Discussion

For physisorbed molecules the image charge screening (Δ*E*_pol.(sub.)_) contribution has been estimated using a point charge at a distance *d* from the image plane (eqn (2)):^57,63^2
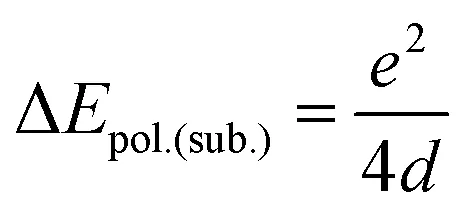


However, while this approach has been successfully applied to planar molecules,^63–65^ it does not accurately capture the magnitude of substrate screening for C_60_. In the latter case, the additional charge is partially delocalized over the spherical molecular geometry and likely preferentially distributed toward the metal-facing side rather than localized at a single point.^47^ Consequently, a simple point-image model assuming a point charge at the molecular centre provides only a crude approximation of the actual screening behaviour (see Fig. S13).

For the present comparison, however, this uncertainty can be partially circumvented: dim 3-fold C_60_ in the islands and C_60_ in the one-dimensional rows adsorb at roughly the same height (see Fig. 4c) and show a 3-fold symmetry in STM (see Fig. 5a and Fig. S10), suggesting similar adsorption geometries. Therefore, the substrate screening term (Δ*E*_pol.(sub.)_) can be approximated to be effectively the same for both species and cancels in a differential analysis. The push-back effect can be treated accordingly, as it also depends on the adsorption height.^66^ This approach allows to focus on relative energy shifts between the two environments, where the remaining differences can be attributed primarily to intermolecular (environmental) screening, as well as variations in the hybridization with neighbouring molecules.^57,63^ Intermolecular interactions are stronger for C_60_ in the close-packed islands than in the intermixed phase, resulting in a larger peak splitting (see Fig. 5) and being attributed to π–π orbital hybridization in the C_60_ islands.^57^

According to Torrente *et al.*,^57^ environmental screening (Δ*E*_env._) depends mainly on the number *N* of surrounding molecules, their polarizability *α*, and the intermolecular distances *R*. Here, we considered Δ*E*_env._ only for adjacent molecules (first-nearest neighbours), due to the *R*^−4^ decay in eqn (3):3
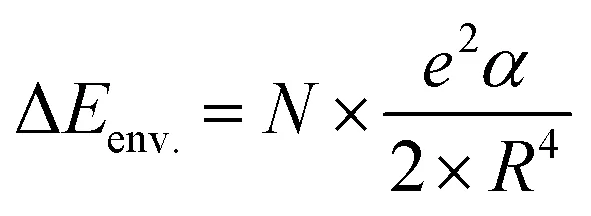


The gas-phase polarizabilities (*α*_0_) for C_60_ and Zn–TAPP were obtained from DFT (see SI). When using these gas-phase polarizabilities, for a C_60_ molecule (*α*_0_ = 549 a.u.) with six C_60_ at *R* = 1.00 nm (see Fig. S12b), eqn (3) yields Δ*E*_env._ = −351 meV. In the intermixed stripe phase, considering two C_60_ and four Zn–TAPP (*α*_0_ = 1024 a.u) and separations of 1.15/1.38/1.47 nm (see Fig. S12c), Δ*E*_env_ is about −228 meV. As a result, despite the larger Zn–TAPP polarizability, increased molecular separations result in a lower net environmental screening (Δ*E*_env._).

The presence of the metallic substrate necessitates correcting the molecular polarizability *α* for image-charge screening according to eqn (4) (see SI, Table S3):^67^4
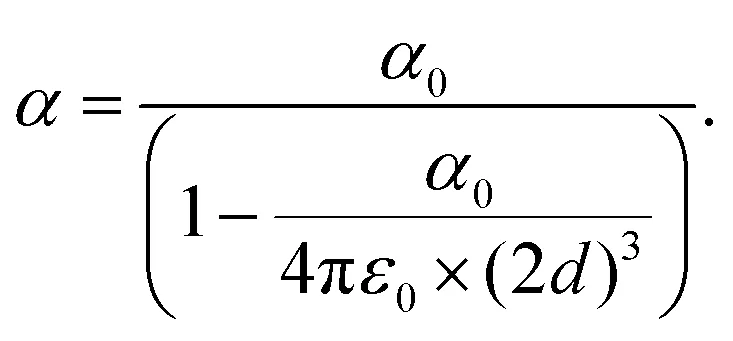


However, this correction requires the use of the simple point-image model resulting in complications for spherical molecules with small adsorption heights (see SI, Fig. S13). Even when locating the image charge at the centre of the molecule (*d* = 0.55 nm), likely underestimating the screening of C_60_ compared to Zn–TAPP, the difference in Δ*E*_env._ between close-packed islands and one-dimensional stripe is minimal (≈40 meV) and cannot account for the observed shift in the d*I*/d*V* spectra (Fig. S14).

After ruling out image-charge screening, push-back effect and estimating minimal differences in environmenl screening, we attribute the observed shift of the LUMOs in the d*I*/d*V* spectra (Fig. 5) to additional charging of C_60_ in the intermixed stripe phase compared to dim 3-fold C_60_ molecules. In this context, intermolecular charge transfer from Zn–TAPP to C_60_ is unlikely, as there is no Zn–TAPP-related occupied orbital sufficiently close in energy to contribute (Fig. S11). Combined with the larger intermolecular distances in the intermixed phase this level alignment also rules out any effects of intermolecular hybridization.^57^ Therefore, we ascribe the excess negative charge accumulation in the C_60_ to increased substrate–molecule charge transfer in the one-dimensional stripes embedded within the otherwise neutral porphyrin scaffold.

DFT calculations for C_60_ monomers and dimers (Fig. S15) support this assignment: charge analysis indicates that C_60_ adsorbed at Ag atom vacancies carries more negative charge than on unreconstructed Ag(111) by about 0.1*e* (see SI Table S4). For monomers, the partial charge increases from ≈0.6*e* on the unreconstructed surface to ≈0.7*e* at a vacancy. Accordingly, C_60_ adsorption energies increase by 0.1–0.3 eV per C_60_ molecule when Ag atom vacancies are introduced.

In close-packed islands, adsorption on reconstructed sites seems to exclusively occur for dim 3-fold C_60_ molecules (resulting in the dim contrast, shown for instance in Fig. 1b and 5a). Since our DFT calculations suggest lower charge transfer for unreconstructed sites, we attribute an even lower degree of charge transfer for the remaining (bright) species in the close-packed islands. Deconvolution of the LCPD (see Fig. 4b and SI) further corroborates enhanced local charge accumulation on C_60_ within the one-dimensional stripe phase. Together, the tunnelling and force spectroscopy analysis demonstrates that C_60_ in the intermixed phase attains a higher negative charge than C_60_ in the close-packed islands.

These findings suggest an electrostatically governed mechanism for SAM formation. In close-packed islands, additional charging of C_60_ is disfavoured by long-range Coulomb repulsion among neighbouring charged molecules. In contrast, in the one-dimensional intermixed phase, larger intermolecular separations and a lower C_60_ coordination number reduce this repulsion, permitting greater charge uptake from the substrate. Therefore, the emergence of one-dimensional C_60_ stripes can thus be understood as a balance between short-range attractive van der Waals dispersion forces, which promote molecular aggregation, and long-range Coulomb repulsion, which inhibits high local charge accumulation. Intercalation into the Zn–TAPP matrix spatially separates the C_60_ molecules, thereby stabilizing a configuration in which C_60_ exhibits a higher degree of charge transfer within the host framework.

## Conclusions

Using C_60_ as a model acceptor, we have shown how electrostatic interactions govern both the assembly and electronic structure of SAMs on Ag(111). KPFS indicates substrate-mediated charging of C_60_, and, after accounting for spatial averaging, reveals enhanced charge accumulation in one-dimensional stripe phases relative to two-dimensional close-packed islands. Consistently, d*I*/d*V* spectroscopy confirms a larger partial occupation of the C_60_ LUMOs in the stripe phase. We attribute this effect to the different lateral electrostatic environments in the two phases. In close-packed islands, strong lateral repulsion raises the chemical potential for additional electron uptake and limits LUMO filling. In the one-dimensional intermixed stripes, increased intermolecular spacing and fewer C_60_ neighbours reduce the electrostatic penalty, enabling higher charging. These results establish molecular packing density and dimensionality as effective levers for tuning charge transfer at metal–molecule interfaces.

This approach suggests that rational variation of the template structure could be used to tune C_60_ charging by systematically modifying the local electrostatic environment in which it is embedded. Extending this concept to templates with different geometries, periodicities, and intermolecular interaction strengths could therefore provide a route to spatially and hierarchically control both C_60_ organization and its degree of charging, offering a general strategy for designing molecular assemblies with coupled structural and electronic functionality.

## Experimental

The Ag(111) single crystal was supplied by SPL and cleaned using alternating cycles of Ne^+^ sputtering (1 keV, 15 min, *p* = 5 × 10^−6^ mbar) and subsequent annealing to 667 K. Zn(ii)–5,10,15,20-tetrakis-(4-aminophenyl)–porphyrin (Zn–TAPP, 98%) was purchased from Porphychem and C_60_ (99.5%) was ordered from Sigma Aldrich. Both molecules were evaporated with a 3-cell Knudsen cell evaporator (Kentax GmbH). Before evaporation on the sample the evaporation rate was determined using a quartz micro balance (QMB). Sub-monolayer coverage was achieved by evaporating ∼5 Hz of both molecules onto the Ag(111) single crystal kept at 300 K. All LT-SPM measurements were conducted at 77 K and ultra-high vacuum (∼5 × 10^−11^ mbar) in a CreaTec Fischer GmbH LT-SPM instrument (bath cryostat with liquid N_2_). Bias voltage (*V*) is given as sample bias with respect to the tip. The surface was analyzed with STM using a Pt/Ir tip or a qPlus sensor with an etched Pt/Ir tip. For the AFM measurements oscillation amplitudes in the range of 0.1–0.3 nm were used. Measurements in the lock-in mode were conducted by adding a sinusoidal voltage modulation (*V*_mod_) at 724 Hz to the bias voltage (*V*).

Models of the Zn–TAPP and C_60_ molecules as the ones shown in Fig. 1b, c and f were built in Avogadro (an open-source molecular builder and visualization tool. Version 1.2.0n https://avogadro.cc/).^68^

DFT calculations were performed in VASP, version 5.4.4.^69,70^ As a model, the Ag(111) surface with 254 Ag atoms (four layers) and 320 Ag atoms (five layers) with the initial separation of 2.889 Å was employed along with D2 dispersion correction, PBE functional and the projector-augmented wave (PAW) method.^71^ In the *z* direction, the cell length was set to 40 Å. Only the gamma point was used in the reciprocal space. First, all models were optimized with the energy cut-off of 250 eV and four layers, bottom two of which were held frozen during optimization to mimic bulk effects. The fullerene molecules were adsorbed in various configurations and only the most stable ones were considered further. The obtained structures were re-optimized with an additional layer of frozen Ag atoms at the cut-off of 300 eV. Final single-point energy evaluations were performed at the energy cut-off of 600 eV. Charges on atoms were analysed using the Bader approach.^72^

## Conflicts of interest

There are no conflicts to declare.

## Supplementary Material

CP-OLF-D6CP03153F-s001

## Data Availability

All raw data are available on the Universität Innsbruck – Data Repository: https://doi.org/10.48323/n51b5-wp225. Supplementary information (SI) is available. See DOI: https://doi.org/10.1039/d6cp03153f.
