# Supplementary material for: Steering interfacial molecular structures by substrate–molecule charge transfer
Source: Phys Chem Chem Phys. 2026 Nov 3. Online ahead of print. doi: 10.1039/d6cp03153f (PMC13728872; doi:10.1039/d6cp03153f)
Supplement: Supplementary file 1 [file CP-OLF-D6CP03153F-s001.pdf]

Supplementary Information (SI) for Physical Chemistry Chemical Physics.
This journal is © the Owner Societies

Supporting information

Steering interfacial molecular structures by substrate-molecule charge transfer

Benjamin Achatz,*[a] Matthias Zeilerbauer,[a] Marco Thaler,[a] Milan Ončák,[b] Shadi Fatayer,[c] and Laerte L. Patera*[a]

[a] *Department of Physical Chemistry, University of Innsbruck, 6020 Innsbruck (Austria).*
E-mail: benjamin.achatz@uibk.ac.at, laerte.patera@uibk.ac.at
[b] *Department of Ion Physics and Applied Physics, University of Innsbruck, 6020 Innsbruck (Austria)*
[c] *Applied Physics Program, Division of Physical Science and Engineering (PSE), King Abdullah University of Science and Technology (KAUST), Thuwal 23955-6900, Saudi Arabia*

Epitaxial relationship of the stripe phase

In Fig. S1 the lattice vectors of the Ag(111) surface have been obtained from the (2√3x2√3)R30° superstructure of C_{60}.[1] Investigation of the epitaxial relationship revealed six distinct orientations of the C_{60} stripes (Fig. S2). Importantly, the C_{60} stripe directions always follow the surrounding porphyrin lattice, as already observed for the stripe phase on Ag(110).[2] In contrast, the stripes do not coincide with the lattice vectors of the (2√3x√3)R30° superstructure, suggesting that the molecular rearrangement upon intercalation of C_{60} into the porphyrin lattice dictates the growth directions of the C_{60} stripes, rather than epitaxial matching of C_{60} with the underlying substrate. To further investigate these structural changes, we compared models of the unit cells of the porphyrin layer and the C_{60} stripe phase extracted from Fig. 1e. The obtained structures can be related to commensurate models (Fig. S3) within an error of below 5% for all lattice parameters. The epitaxial relationships of the commensurate structures with respect to the basis vectors of the Ag(111) substrate are given by the matrices M in Fig. S3. The models shown in Fig. S3 reveal that all porphyrins are oriented along the [-101] direction of the Ag(111) substrate. C_{60} intercalation requires an expansion of the porphyrin lattice which can be described as a translation of two porphyrins along the [0-11] direction, by twice the lattice constant of Ag(111) (red arrow in Fig. S3). Diffraction measurements would be required to confirm these commensurate structures, but they lie outside the scope of this study.

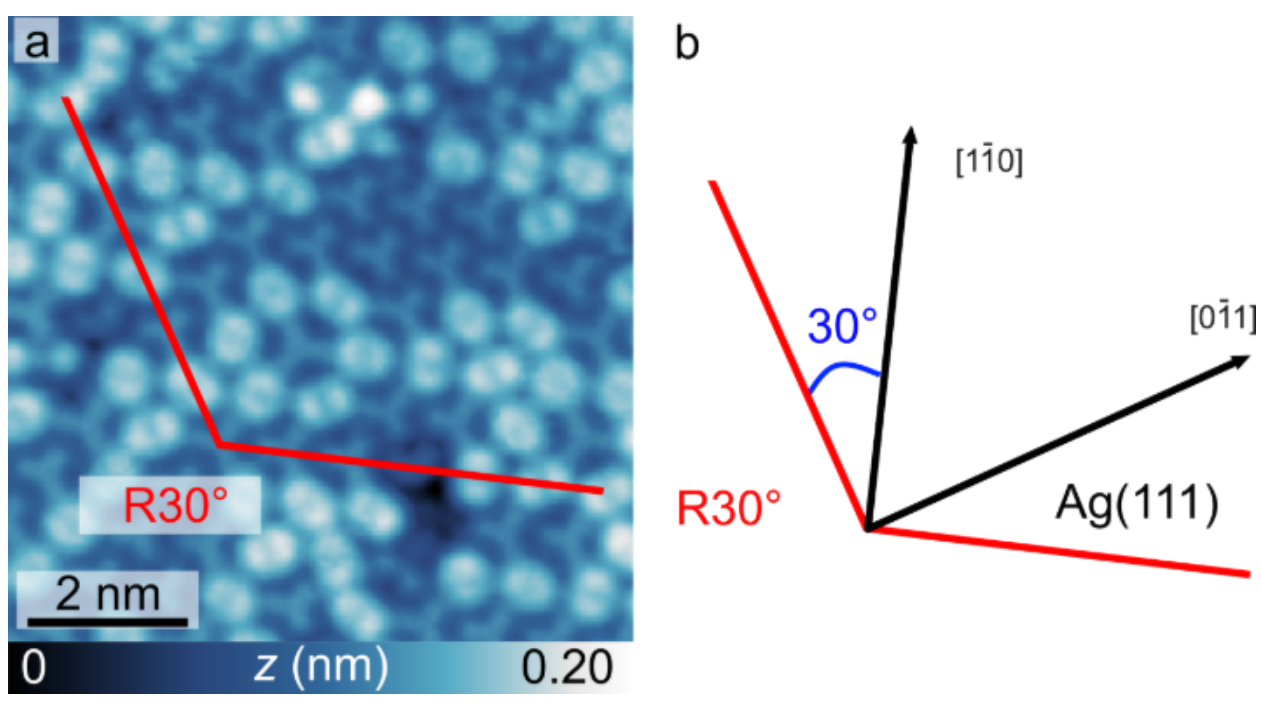

Fig. S1. (2√3x2√3)R30° C_{60} superstructure on Ag(111). (a) Constant-current STM image of a C_{60} island on Ag(111) (lattice vectors indicated in red). (b) Inferred crystallographic directions on the Ag(111) surface (black). Measurement parameters: (a) I = 10 pA, V = 0.2 V.

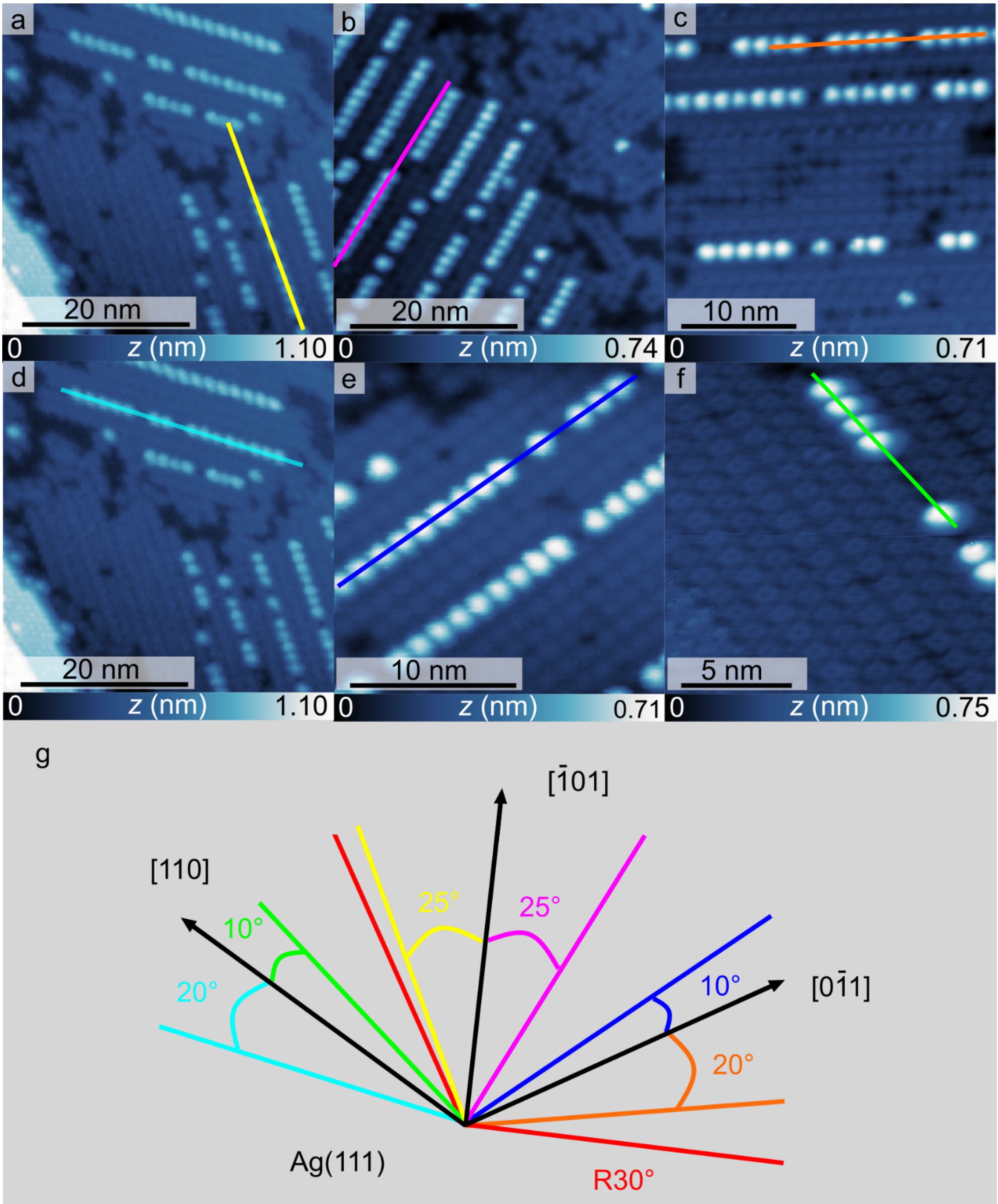

Fig. S2. C_{60} stripes growth directions. (a-f) Constant-current STM image of C_{60} chains on Ag(111) (g) Inferred growth direction of the C_{60} chain in (a-f) compared to the lattice on the Ag(111) surface (black) and the (2√3x2√3)R30° C_{60} superstructure (red). Measurement parameters: (a,d) I= 8 pA, V= 1.0 V (b,e) I= 5 pA, V= 0.6 V (c) I= 20 pA, V= 0.5 V (f) I= 6 pA, V= 0.4 V.

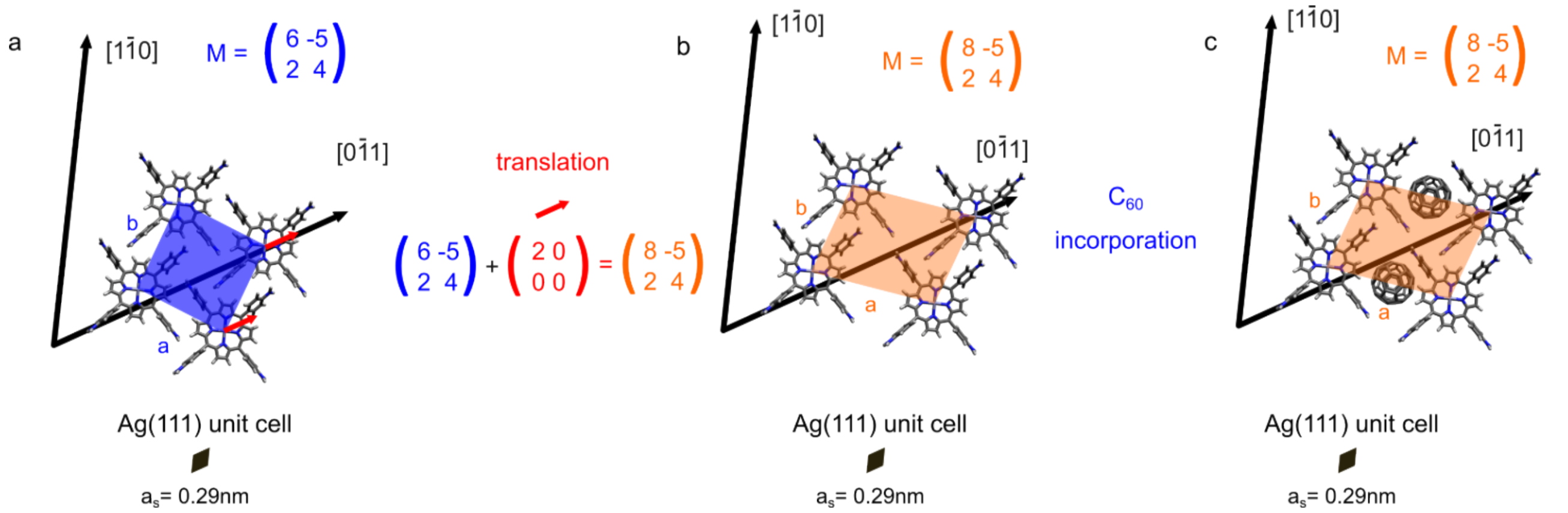

Fig. S3. Structural changes in the porphyrin lattice during C_{60} intercalation for commensurate models. Commensurate model of (a) pristine Zn-TAPP lattice, (b) Zn-TAPP lattice after translation of two porphyrins and (c) Zn-TAPP lattice hosting one C_{60} per unit cell. Epitaxial relationship to the Ag(111) basis vectors is given by the matrices M. Model based on overlays of Fig. 1e.

KPFS

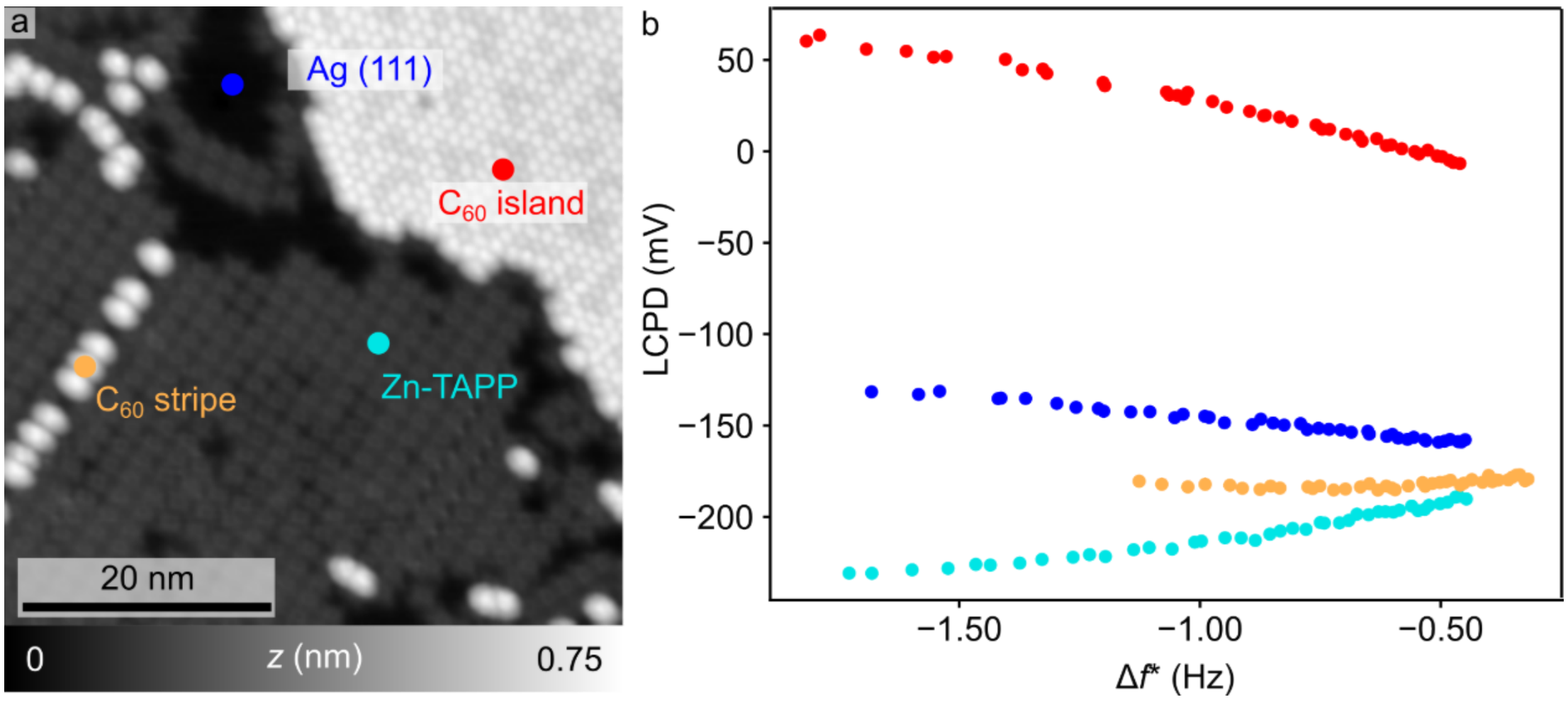

Fig. S4. Height-dependent KPFS. (a) Constant-current STM image acquired after annealing to 428 K for 3 h. (b) Fitted LCPD values acquired at the positions marked in (a) as a function of Δf^*. Measurement parameters: (a) I = 20 pA, V = 0.5 V; (b) Feedback opened on the marked positions at I = 5 pA, V = 0.6 V, A = 0.3 nm.

Lateral Resolution in KPFM

The lateral resolution of KPFM is limited due to the averaging of long-range electrostatic forces. To estimate the lateral resolution in our experiments, we acquired KPFS spectra at different tip-sample distances across the interface between C_{60} and Zn-TAPP islands (marked by an arrow in Fig. S5a). Figure S5b shows the decreasing LCPD contrast between the C_{60} island and adjacent Zn-TAPP layer with increasing tip height (z). The LCPD profiles were fitted with an error function (Fig. S5c) and the resolution was estimated by calculating the full width half maximum (FHWM) of the gaussian component from its standard deviation (σ).

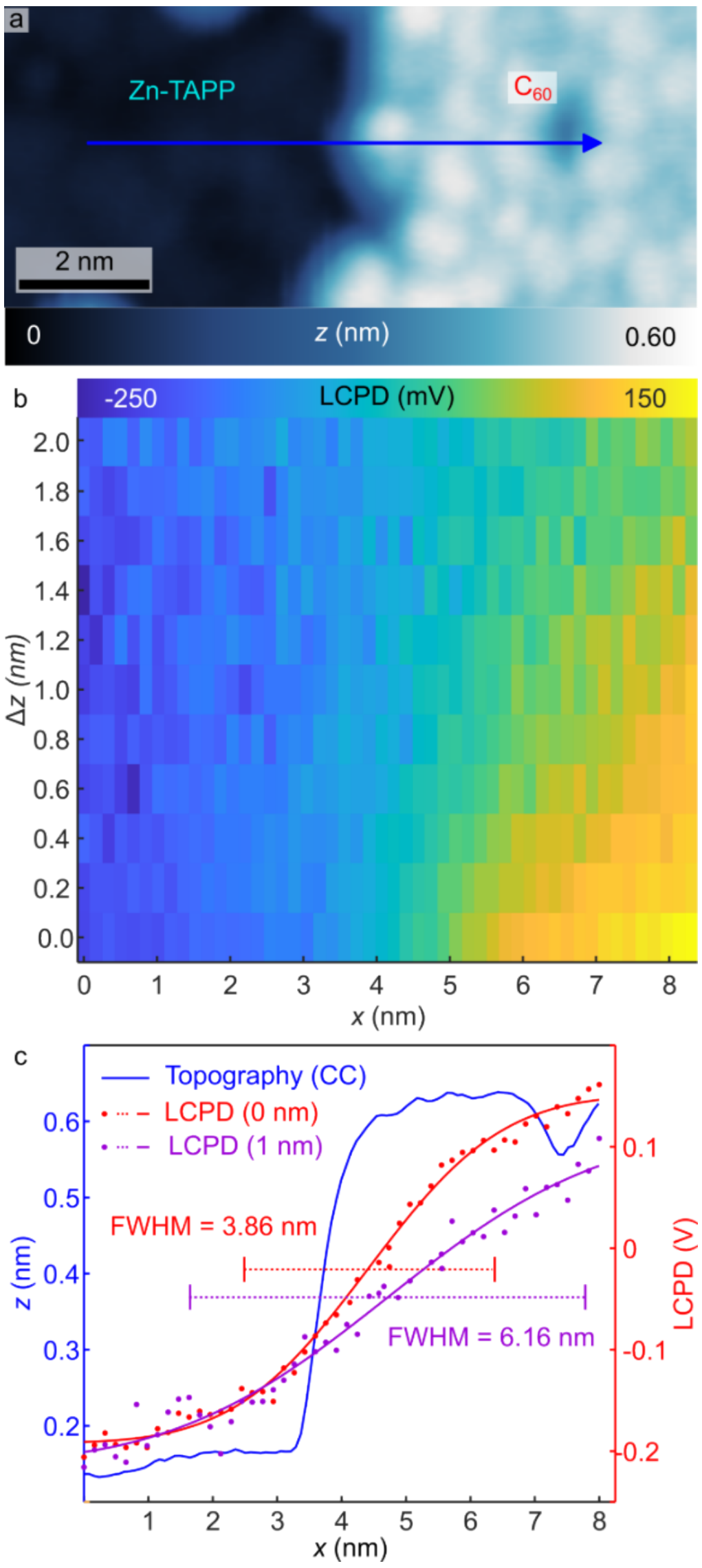

Fig. S5. Tip-height dependence of LCPD spatial resolution. (a) Constant-current STM image. (b) LCPD acquired across a step edge, indicated by the arrow in (a), at different tip-sample distances in increments of 0.2 nm. (c) STM topographic line profile extracted form image (a) and extracted LCPD at $z = 0$ nm (FWHM = 3.86 nm $\sigma = 1.64$ nm) and $z = 1$ nm (FWHM = 6.16 nm and $\sigma = 2.61$ nm). Measurement parameters: (a) tunneling current $I = 10$ pA, sample bias voltage $V = 0.25$ V. (b-c) Feedback opened at $I = 10$ pA, $V = 0.25$ V, $A = 0.3$ nm.

To estimate the intrinsic LCPD of the C_{60} stripe from the measured LCPD, we modelled the measurement as the convolution of the surface dipole variation with a point-spread function (PSF).[3] A gaussian PSF and a double step function were used for the deconvolution. This simplified modeling of the PSF and the intrinsic LCPD qualitatively describes the smeared out contrast in KPFS. The width of the double step function was set to 0.7 nm, which roughly corresponds to the size of a C_{60} molecule. By fitting the experimental data shown in Fig. S6a, we determined a FWHM (1.85±0.15 nm) of the PSF ($FWHM_{PSF}$) – which is considerably smaller than the one determined for the gaussian component of the error function in Fig. S6 (FWHM = 3.86±0.18 nm). This value of the $FWHM_{PSF}$ can be then used to determine the total contribution to the measured LCPD of the C_{60} molecules in a one-dimensional stripe phase. We modeled the C_{60} stripe phase as seven two-dimensional disks with a diameter of 0.7 nm, arranged collinearly with a center-to-center spacing of 1.47 nm, to emulate the C_{60} stripe-phase assembly (Fig. S6b). The object was then convolved with a Gaussian point-spread function (PSF) using the fitted $FWHM_{PSF}$. We then evaluated, at the position of the central disk, the contribution of each individual disk to the deconvolved signal (Fig. S3b). Only the center molecule and its two nearest neighbors contribute appreciably (see Fig. S6c), indicating that measured LCPD in Fig. 4b reflects just 12.8% of the intrinsic stripe contrast (yielding a renormalization factor of 7.8±0.6). An additional profile acquired at $z = 0.1$ nm (Fig. S7) yielded a similar renormalization factor of 8.2±1.0. The increased error on the renormalization factor can be attributed to lower signal to noise ratio in KPFS upon retracting the tip. While these deconvolutions do not provide quantitative information about the charge in the C_{60} stripes they can be used to compare the magnitude of the LCPD with the two-dimensional C_{60} islands. In contrast to the stripe case, the C_{60} islands have lateral dimensions that are much larger than the $FWHM_{PSF}$ (see Fig. S8a). Therefore, the contrast measured 5 nm inside the island is effectively insensitive to PSF convolution (Fig. S8b). According to this simplified model the deconvolved LCPD of the stripe phase is

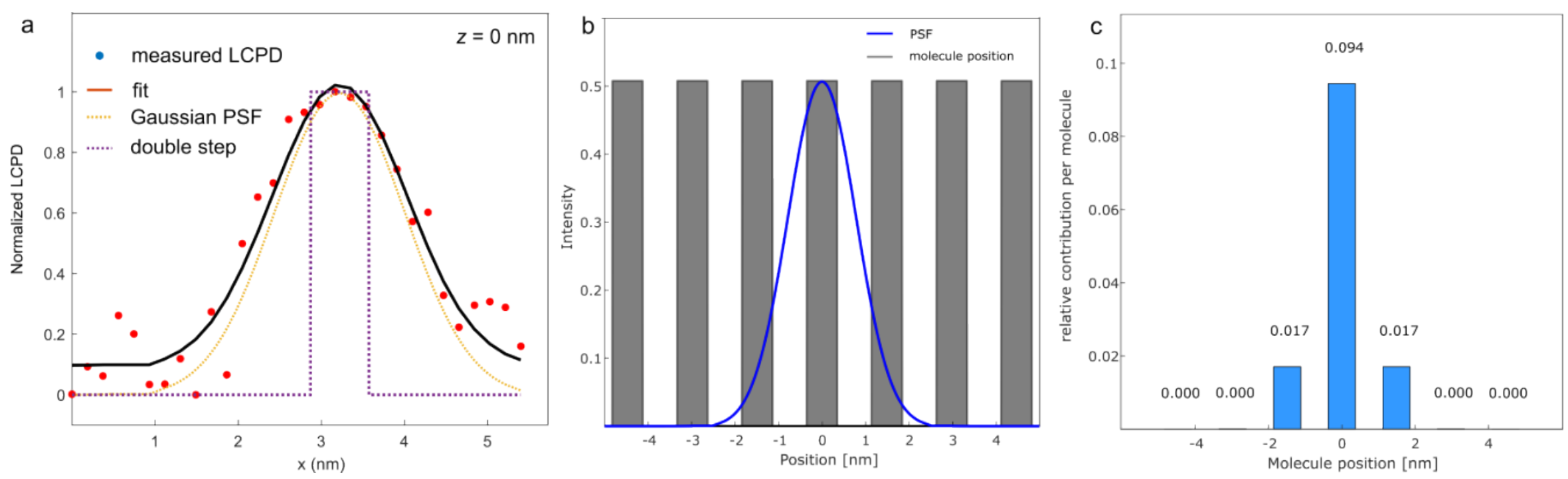

Fig. S6. Deconvolution of the measured LCPD in a C_{60} stripe phases, shown in Fig. 4b. (a) Double step functions with a width of 0.7 nm have been convolved with a gaussian point spread function (PSF) to fit the experimental data taken along the profile shown in Fig. 4. (b) The Gaussian PSF from the fit ($FWHM_{PSF}$ = 1.85±0.15 nm) was used to determine the contribution of the molecules in a 1-dimensional stripe with center-center distances of 1.47 nm. (c) Average contribution of the individual molecules to the total signal.

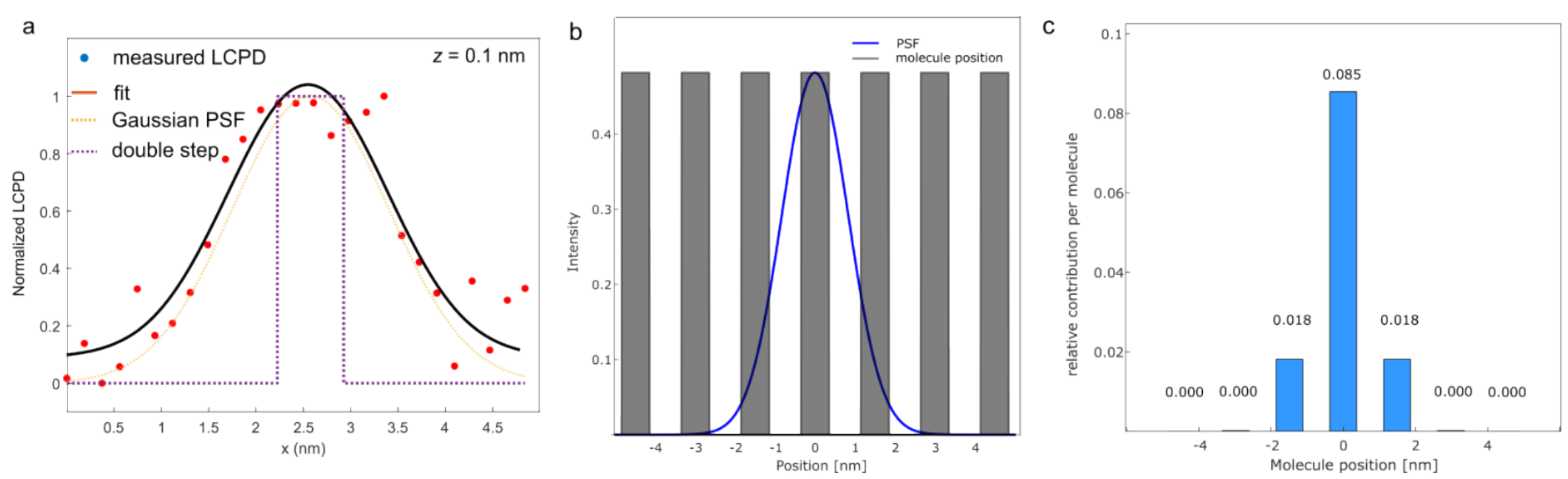

Fig. S7. Deconvolution of the measured LCPD in a C_{60} stripe phases, along the profile in Fig. 4b after retracting the tip by 0.1 nm. (a) Double step functions with a width of 0.7 nm have been convolved with a gaussian point spread function (PSF) to fit the experimental data taken along the profile shown in Fig. 4. (b) The Gaussian PSF from the fit ($FWHM_{PSF}$ = 1.95±0.23 nm) was used to determine the contribution of the molecules in a 1-dimensional stripe with center-center distances of 1.47 nm. (c) Average contribution of the individual molecules to the total signal.

around eight times larger than the measured LCPD, supporting the assumption that averaging effects heavily suppresses the contrast for one-dimensional stripe phases, compared to the islands.

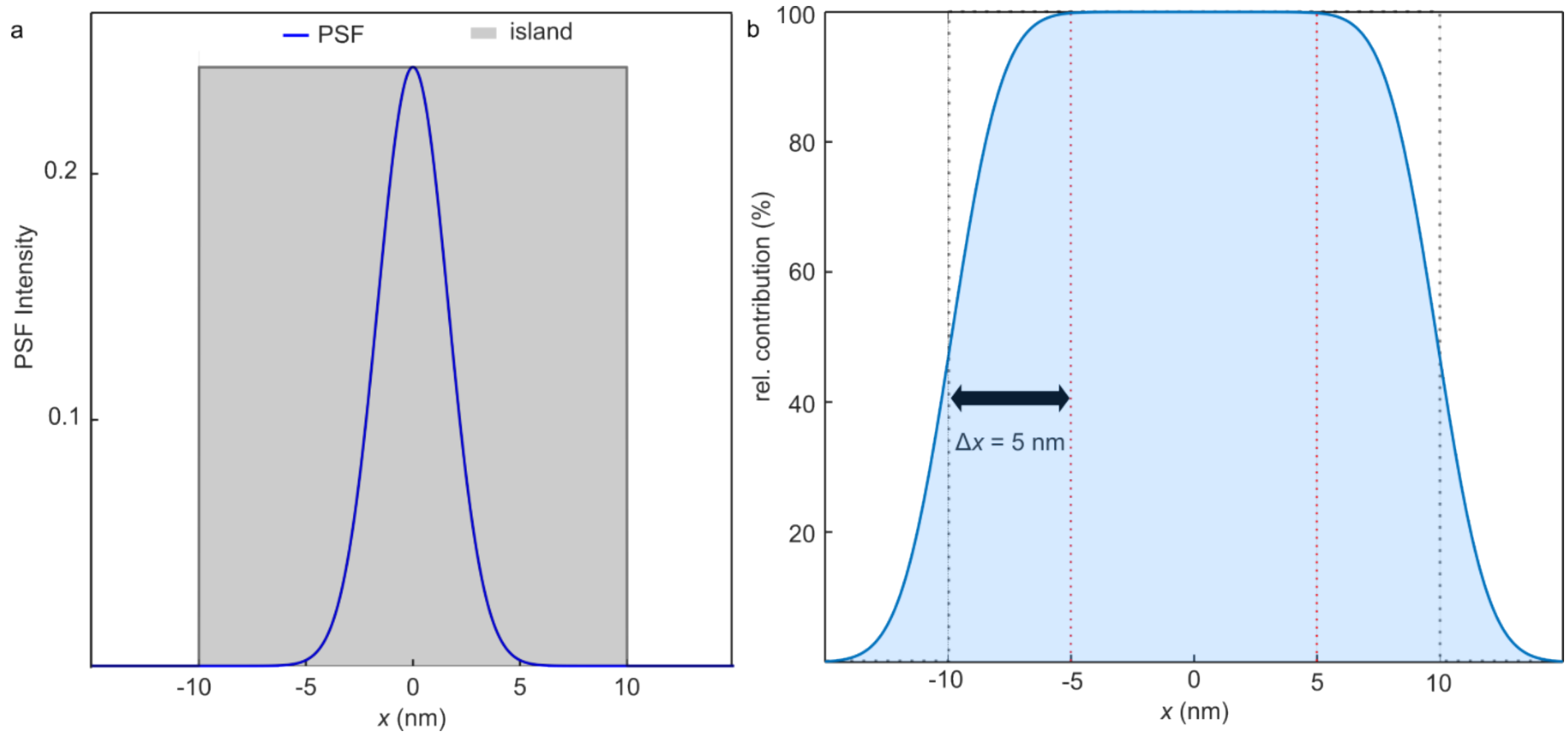

Fig. S8. Deconvolution of the measured LCPD for a 20 nm island. (a) The Gaussian PSF from the fit in Fig. S3a (FWHM = 3.86 nm) was used to determine the contribution of a 20 nm island. (b) Contribution of the island as a function of the tip position.

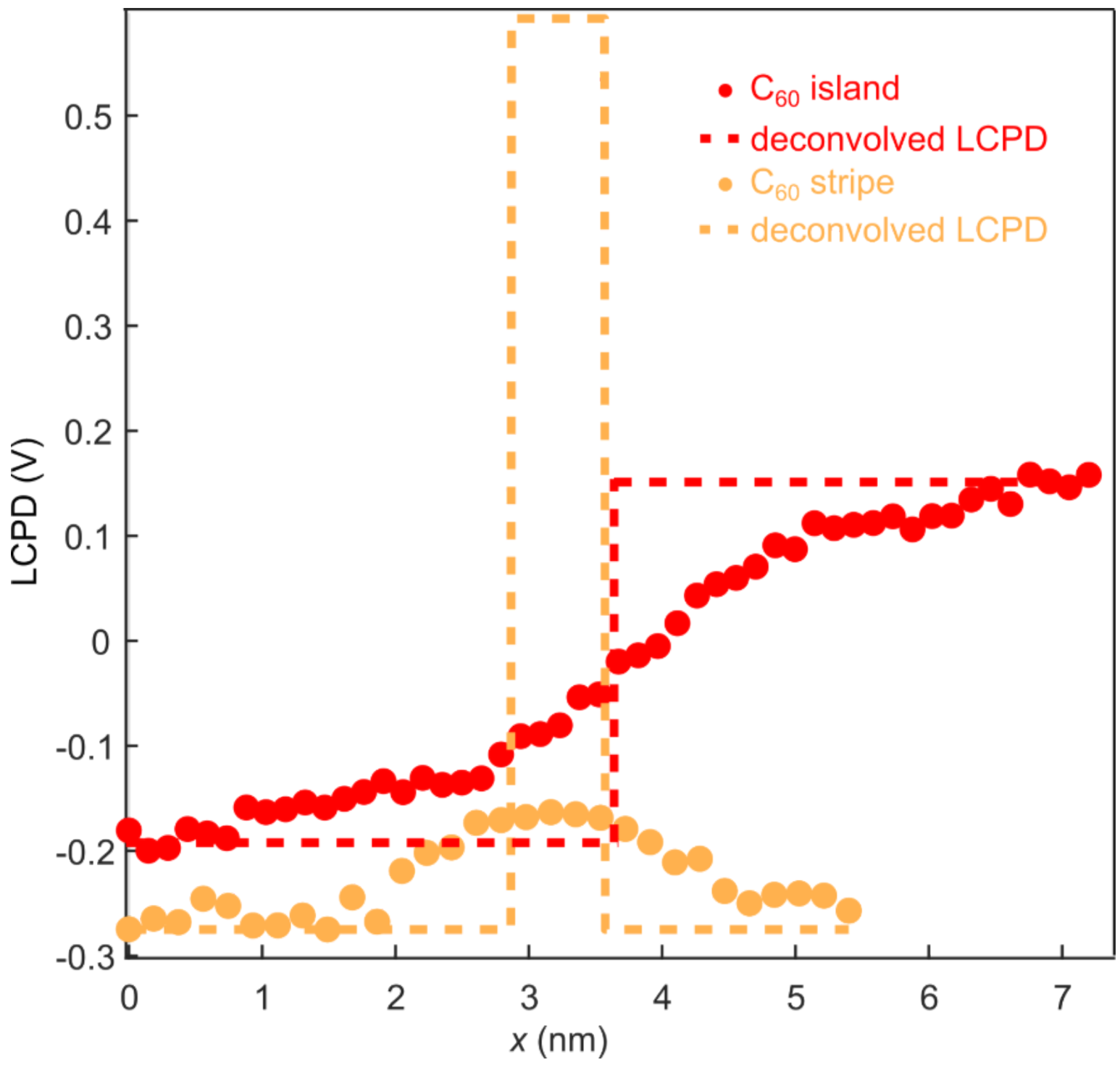

Fig. S9. Deconvolved LCPD for the C_{60} stripe phase and island. LCPD acquired with constant height feedback along the path indicated by the arrows in Fig. 4a: across the edge of a C_{60} island (red) and a C_{60} stripe (orange). Feedback opened at I = 10 pA, V = 0.25 V, Δz = 0.0 nm, A = 0.3 nm.

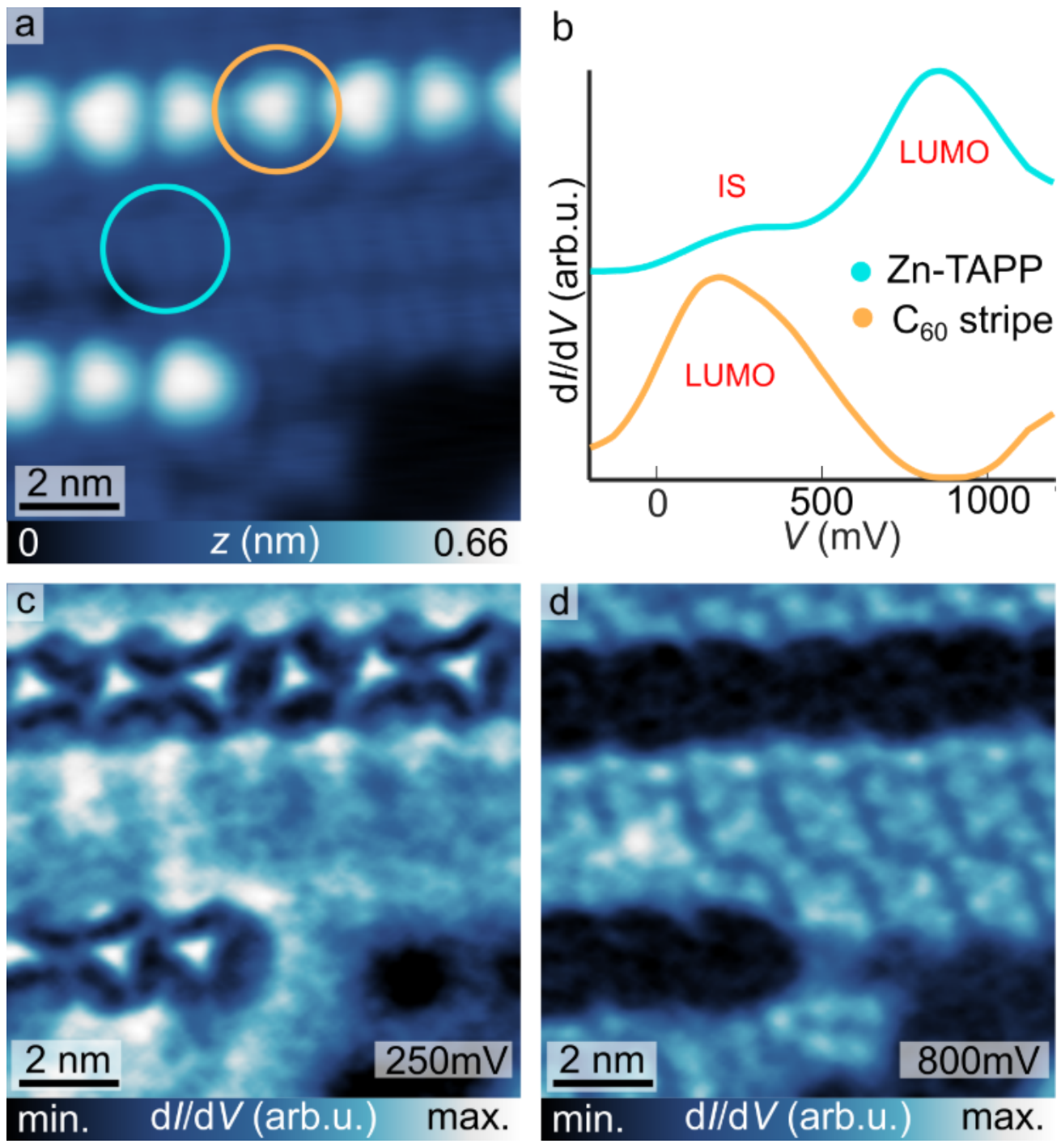

Fig. S10. (a) Constant-current STM image of the stripe phase. (b) d*I*/d*V* spectra acquired on C_{60} and Zn-TAPP. (c-d) Constant-current d*I*/d*V* maps acquired at V = 250 mV and 800 mV. Measurement parameters: (a) tunneling current I = 50 pA, sample bias voltage V = 0.25 V. (b) Feedback opened at I = 50 pA, V = 1.20 V (c) I = 50 pA, V = 0.25 V. (e) I = 50 pA, V = 0.80 V. Sample bias voltage modulation V_{mod} = 0.04 V_{rms}.

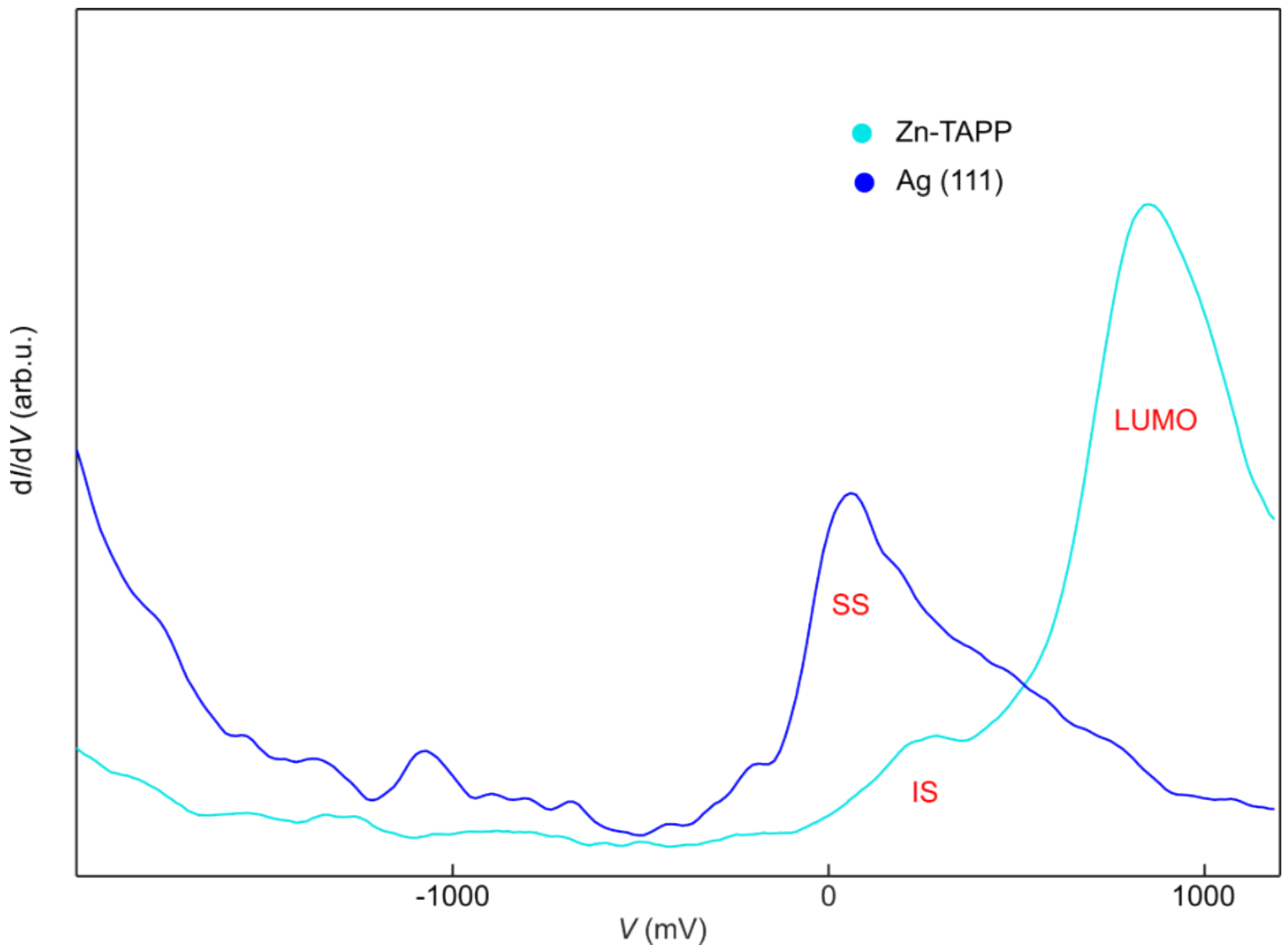

Fig. S11. dI/dV spectra acquired on Zn-TAPP and the Ag(111) substrate. Surface state (SS) of Ag(111), the LUMO and an interface state (IS) on Zn-TAPP are indicated. Measurement parameters: Feedback opened at I = 60 pA, V = 0.80 V. Sample bias voltage modulation V_{mod} = 0.04 V_{rms}.

Table S1: Fit parameters for the spectra shown in Figure 5b,c,e,f.

Spectrum	C_{60} dim 3-fold	C_{60} bright 2-fold	C_{60} bright 3-fold	C_{60} stripe
Amp. 1	1.259	2.215	1.950	4.822
Center 1	0.135	0.233	0.331	0.149
Width 1	0.102	0.109	0.144	0.145
Amp. 2	3.634	1.910	1.215	0.000
Center 2	0.471	0.431	0.600	0.500
Width 2	0.152	0.159	0.103	0.100
Amp. 3	1.405	1.534	0.939	3.988
Center 3	0.810	0.727	0.788	0.420
Width 3	0.119	0.180	0.153	0.143
Amp. 4	2.294	3.264	2.534	1.733
Center 4	1.286	1.281	1.242	1.239
Width 4	0.180	0.180	0.168	0.163
Baseline	1.038	0.517	-0.045	1.790

Table S2: Estimated degree of charge transfer from the spectra shown in Figure 5b,c,e,f. Degree of charge transfer estimated from the areas of three gaussian functions below 0 V (according to equation 1)

Spectrum	C_{60} dim 3-fold	C_{60} bright 2-fold	C_{60} bright 3-fold	C_{60} stripe
Area 1	0.321	0.608	0.704	1.755
Area 2	1.388	0.763	0.315	0.000
Area 3	0.418	0.692	0.359	1.434
Total Area	2.127	2.063	1.378	3.189
% Below Zero	1.462	0.621	0.554	8.437
n_{e^-} / C_{60}	0.088	0.037	0.033	0.506

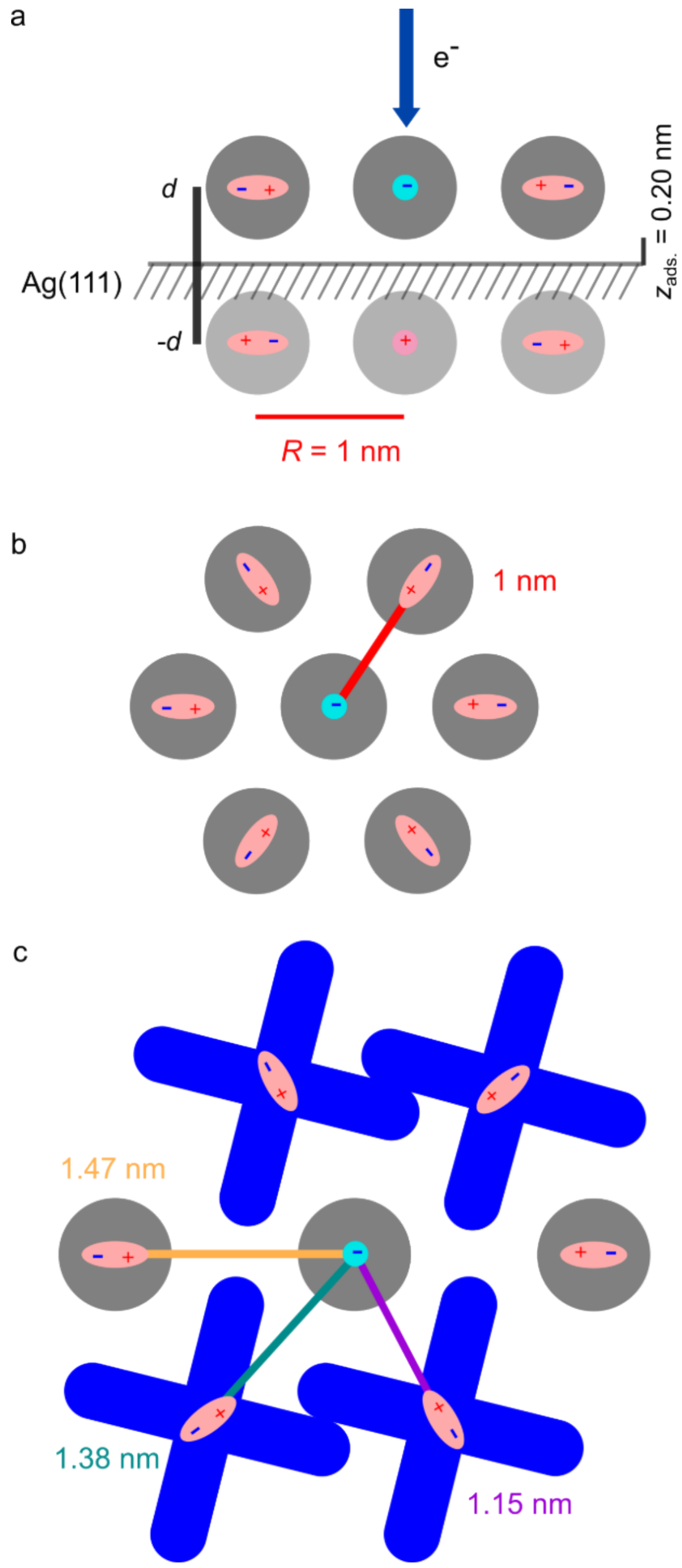

Fig. S12. (a) Schematic drawing of the image charge screening in the underlying Ag(111) substrate and environmental screening. Sketches of the C_{60} molecular arrangements in close packed islands (b) and in the stripe phase (c).

The gas-phase polarizabilities of C_{60} and Zn–TAPP were computed with DFT as implemented in ORCA 6.1.0[4-6]. Prior to the polarizability calculations, the molecular geometries were optimized using the hybrid B3LYP functional[7,8] together with the def2-TZVP basis set[9] and the def2/J auxiliary basis[10,11], employing the RIJCOSX approximation[11] and Grimme's D3 dispersion correction with Becke–Johnson damping (D3BJ[5]). The static polarizabilities were then evaluated at the B3LYP level using the diffuse-augmented def2-TZVPD basis set[9,12,13], in combination with the def2/J auxiliary basis, the RIJCOSX approximation, and the D3BJ dispersion correction.

To estimate the environmental screening – including the image charge correction for the polarizabilities[14] – in the two-dimensional island, we treated the six nearest C_{60} molecules as the local environment. Regarding the one-dimensional stripe phase, we considered that a C_{60} has four Zn-TAPP molecules and two C_{60} as its nearest neighbors. To take the presence of the metallic substrate into account, the anisotropic polarizabilities obtained from DFT calculations (Table S3) were corrected using equation 4. For the Zn-TAPP molecules placing a point charge in the center of the molecule serves as a reasonable approximation (Fig. S12). We assumed an adsorption height ($z_{ads.}$) similar to that of Zn(II) 5,10,15,20-tetraphenylporphyrin (Zn-TPP), which was previously determined by DFT calculations (Table S3).[15] Owing to the near-spherical nature of C_{60} and the polarization effects introduced by the substrate,[16] treating the system with a simple point-charge model is not reliable. According to previous works on C_{60}, when using point charges they should be placed at the bottom of the molecule.[17] However, due to the small adsorption heights of C_{60} on Ag(111),[18] further complications arise: when the charge and image charge get too close to each other the function diverges (Fig. S13). Importantly, even when placing the point charge at the center of the C_{60} molecule, the environmental screening – calculated according to equation 3 – is similar for both phases (Fig. S14). Furthermore, in this simplified model the image charge screening ($\Delta E_{pol.(sub.)}$) is the dominant contribution (Fig. S14).

Table S3. Gas-phase polarizabilities and adsorption heights ($z_{ads.}$).

/	**Zn-TAPP**	**C_{60} dim**
α_0 (a.u.)	1024	549
$z_{ads.}$ (nm)	0.33[15]	0.20[18]

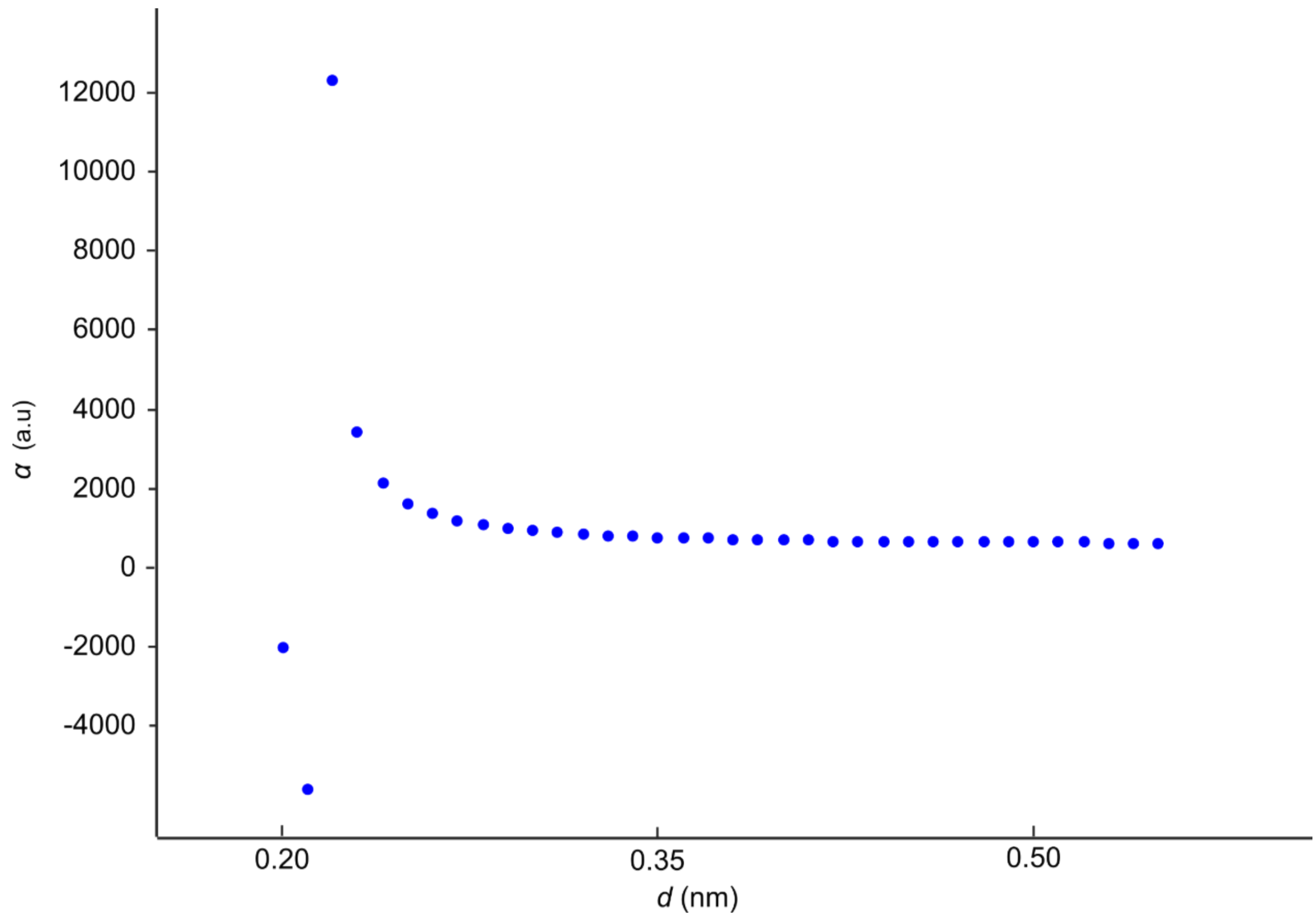

Fig. S13. Distance dependence of the corrected polarizability (α$_{corr.}$) for a point charge.

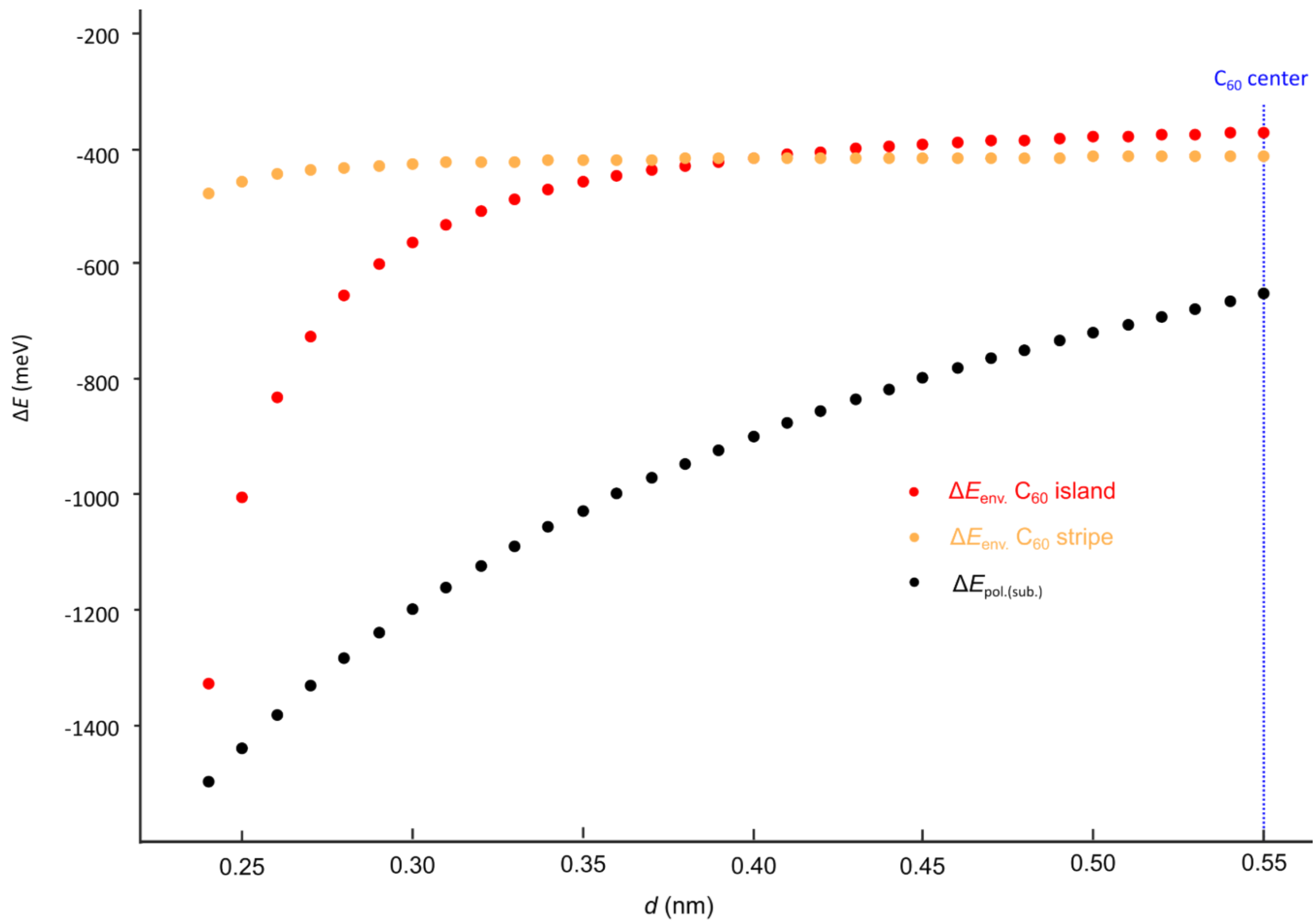

Fig. S14. Distance dependence of image charge screening (Δ$E_{pol.(sub.)}$) and the environmental screening (Δ$E_{env.}$) for a point charge the six nearest C_{60}.

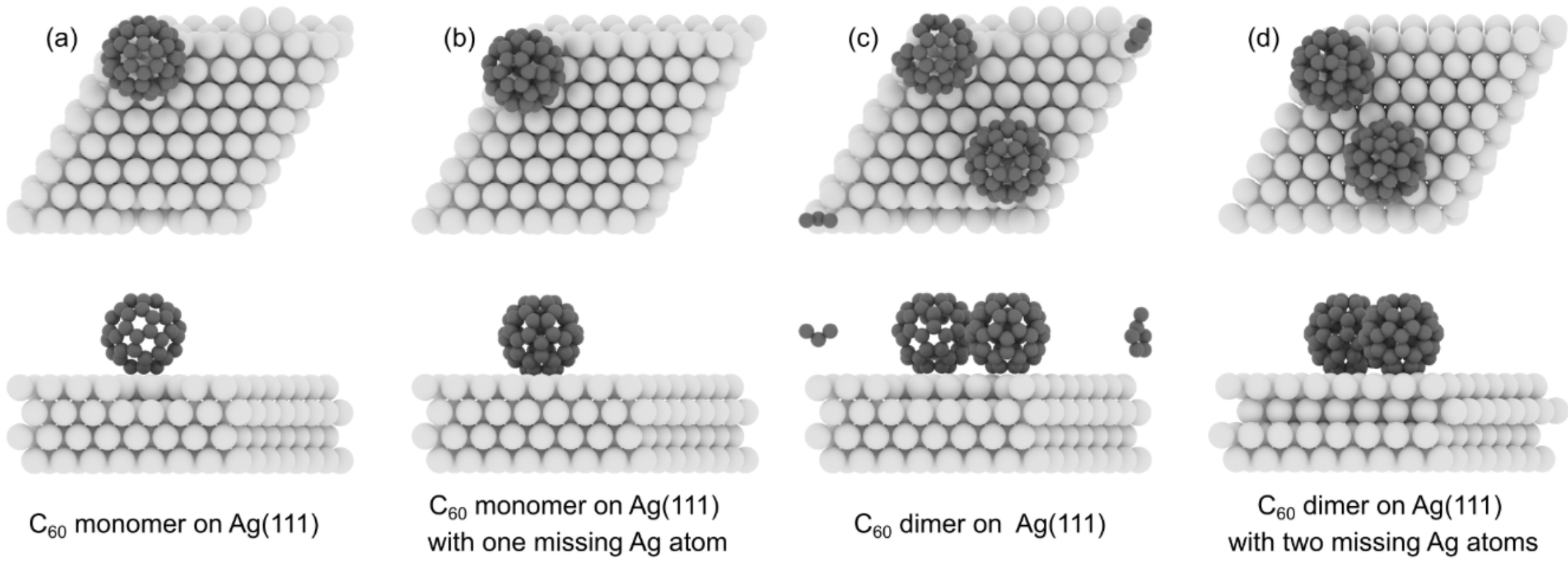

Fig. S15. DFT geometry optimized structures for C_{60} monomers and C_{60} dimers on 4 layers of Ag(111). (a,c) C_{60} on unreconstructed Ag(111). (b,d) Single Ag atom vacancies underneath the C_{60} molecules.

Table S4. Charges and adsorption energies from DFT calculations. For adsorption energies, results with two different energy cut-offs are provided, see the text.

Model	C_{60} monomer unreconstructed	C_{60} monomer reconstructed	C_{60} dimer unreconstructed	C_{60} dimer reconstructed
q	−0.65	−0.74	−0.59/−0.59	−0.73/−0.73
E_{ads} / eV (E_{cut} = 600 eV)	2.9	3.0	5.5	6.2
E_{ads} / eV (E_{cut} = 300 eV)	2.9	3.1	5.6	6.3

References

1 X.-Q. Shi, M. A. Van Hove and R.-Q. Zhang, *J Mater Sci*, 2012, **47**, 7341–7355.

2 F. Sedona, M. Di Marino, A. Basagni, L. Colazzo and M. Sambi, *J. Phys. Chem. C*, 2014, **118**, 1587–1593.

3 56 G. Elias, T. Glatzel, E. Meyer, A. Schwarzman, A. Boag and Y. Rosenwaks, *Beilstein J. Nanotechnol.*, 2011, **2**, 252–260.

4 F. Neese, *WIREs Comput. Mol. Sci.*, 2025, **15**, e70019.

5 S. Grimme, S. Ehrlich and L. Goerigk, *J. Comput. Chem.*, 2011, **32**, 1456–1465.

6 A. D. Becke, *J. Chem. Phys.*, 1993, **98**, 5648–5652.

7 C. Lee, W. Yang and R. G. Parr, *Phys. Rev. B*, 1988, **37**, 785–789.

8 F. Weigend and R. Ahlrichs, *Phys. Chem. Chem. Phys.*, 2005, **7**, 3297–3305.

9 F. Weigend, *Phys. Chem. Chem. Phys.*, 2006, **8**, 1057.

10 K. Eichkorn, F. Weigend, O. Treutler and R. Ahlrichs, *Theor. Chem. Acc.*, 1997, **97**, 119–124.

11 S. Kossmann and F. Neese, *Chem. Phys. Lett.*, 2009, **481**, 240–243.

12 D. Rappoport and F. Furche, *J. Chem. Phys.*, 2010, **133**, 134105.

13 D. Rappoport, *J. Chem. Phys.*, 2021, **155**, 124102.

14 C. M. Teodorescu, *Phys. Chem. Chem. Phys.*, 2015, **17**, 21302–21314.

15 W. Hieringer, K. Flechtner, A. Kretschmann, K. Seufert, W. Auwärter, J. V. Barth, A. Görling, H.-P. Steinrück and J. M. Gottfried, *J. Am. Chem. Soc.*, 2011, **133**, 6206–6222.

16 T. Chutora, J. Redondo, B. De La Torre, M. Švec, P. Jelínek and H. Vázquez, *Beilstein J. Nanotechnol.*, 2017, **8**, 1073–1079.

17 P. Amsalem, J. Niederhausen, A. Wilke, G. Heimel, R. Schlesinger, S. Winkler, A. Vollmer, J. P. Rabe and N. Koch, *Phys. Rev. B*, 2013, **87**, 035440.

18 H. I. Li, K. Pussi, K. J. Hanna, L.-L. Wang, D. D. Johnson, H.-P. Cheng, H. Shin, S. Curtarolo, W. Moritz, J. A. Smerdon, R. McGrath and R. D. Diehl, *Phys. Rev. Lett.*, 2009, **103**, 056101.
